# Global burden and health inequality of nutritional deficiencies from 1990 to 2019

**DOI:** 10.3389/fnut.2024.1470713

**Published:** 2024-09-25

**Authors:** Yang Yu, Hui Li, Nan-xi Hu, Xiao-hua Wu, Xin-yi Huang, Hong-tao Lin, Kai-li Yu, Jin-luan Li

**Affiliations:** ^1^Department of Quality Control, Clinical Oncology School of Fujian Medical University, Fujian Cancer Hospital, Fuzhou, China; ^2^Department of Radiation Oncology, Clinical Oncology School of Fujian Medical University, Fujian Cancer Hospital, Fuzhou, China

**Keywords:** nutritional deficiencies, Global Burden of Disease, incidence, prevalence, DALYs, mortality, health inequality

## Abstract

**Introduction:**

Nutritional deficiencies (NDs) manifest in various forms and are widespread globally. However, a systematic evaluation of the epidemiology of NDs across all causes and age groups in different countries and regions has not been conducted.

**Materials and methods:**

This study aimed to utilize data from the 2019 Global Burden of Disease (GBD) study to assess the burden and trends of NDs, including their incidence, prevalence, mortality, and disability-adjusted life years (DALYs). Additionally, the study evaluated health inequalities at global, regional, and national levels from 1990 to 2019.

**Result:**

In 2019, the age-standardized incidence rate of NDs was 2,207.71 per 100,000 individuals (95% UI 1,863.04–2,604.67), and the age-standardized DALYs (ASR-DALYs) was 680.12 per 100,000 individuals (95% UI 507.21–894.89). Among the causes of NDs, dietary iron deficiency had the highest ASR-DALYs and exhibited minimal variation. Children under the age of 5 years faced the greatest risk of NDs. Sex disparity was evident, with males having lower rates than females. Although the gap in the burden of NDs between regions classified as poor and wealthy decreased, disparities persist.

**Conclusion:**

These findings provide critical insights for the development of global health strategies aimed at mitigating NDs and may guide policymakers in implementing effective and economically viable interventions.

## 1 Introduction

There is no universal consensus on the definition and clinical evaluation of nutritional deficiencies (NDs); however, NDs typically fall into two primary categories: protein–energy malnutrition and micronutrient deficiencies (such as iron, vitamin A, and iodine) (1). Protein–energy malnutrition represents a state of disrupted catabolism stemming from metabolic disturbances or inadequate nutrition, leading to chronic illnesses, hypoglycemia, hypothermia, and severe infections (2). Micronutrient deficiencies can result in vulnerability to infections, congenital abnormalities, vision impairment, impaired growth, cognitive deficits, reduced academic and work performance, and even death, affecting people globally (3). NDs affect approximately 33% of the global population, manifesting as wasting, stunting, vitamin and mineral deficiencies, overweight, and obesity (4). In addition to health repercussions, malnutrition substantially hinders the social and economic development of the nations. The annual economic cost of lost productivity and healthcare due to malnutrition is estimated at approximately USD 3.5 trillion (5).

Recent research in this area often focused on groups that bear a greater burden, such as children under 5 years of age, pregnant and postpartum women, and older adults (6–12). Some studies have concentrated on particular conditions, such as iodine deficiency (13), vitamin A deficiency (14, 15), protein–energy malnutrition (16), acute malnutrition (17), and other micronutrient deficiencies (18). Significant regional differences in nutritional status and cross-country inequalities in the incidence of NDs are widely acknowledged. Unfortunately, no existing study has comprehensively evaluated the epidemiology of NDs across all causes and age groups throughout various geographical locations.

Therefore, this study aimed to evaluate the burden and trends of NDs, encompassing metrics such as incidence, prevalence, mortality, disability-adjusted life years (DALYs), and health inequality. This evaluation spans across various geographical locations over the two-decade period from 1990 to 2019. The goal was to enhance public understanding of strategies for the prevention and management of NDs as well as to provide evidence-based guidance for health policy development.

## 2 Materials and methods

### 2.1 Data source

The Global Burden of Disease (GBD) database is a comprehensive and standardized repository of health and disease information, which provides estimates of the burden of diseases and injuries across the global population. This database is developed and maintained by the Institute for Health Metrics and Evaluation (IHME) at the University of Washington (19). In addition, this assessment was based on the latest and accessible epidemiological survey data and enhanced by optimized standardized methodologies. Data for this assessment were retrieved from the GBD 2019 database using the Global Health Data Exchange (GHDx) query tool, accessible at http://ghdx.healthdata.org/gbd-results-tool (20).

### 2.2 Main metrics

This investigation utilized four metrics to evaluate the disease burden: incidence, prevalence, mortality, and DALYs. To adjust for differences in age structure across disparate populations, the rates of these indicators were computed by dividing each respective measure by the population size. Age-standardized estimates were employed to harmonize these metrics to a standard global population, thereby enabling population-to-population comparisons of disease burden (21).

### 2.3 Global burden and annual change

To assess the temporal trends of the age-standardized incidence rates (ASIR), age-standardized DALYs rates (ASR-DALYs), age-standardized prevalence rates (ASPR), and age-specific mortality rates (ASMR) over the past 30 years, the estimated annual percentage change (EAPC) was calculated (22, 23). The equation used for this linear regression model was *Y* = *α* + *βX* + *ε*, where *X* represents the calendar year, *Y* stands for the natural logarithm of each age-standardized indicator, and *ε* indicates the error term. EAPCs were calculated as 100 × (exp(β) – 1). Additionally, the corresponding 95% confidence interval (CI) for these estimates using the previously mentioned linear regression model was computed. A 95% CI that falls below zero suggests a declining trend, whereas a CI above zero indicates an increasing trend. A CI that includes zero signifies a stable trend.

### 2.4 Decomposition analysis

A decomposition methodology was employed to calculate the contribution of NDs, their incidence, prevalence, DALYs, and mortality based on aging, population growth, and epidemiological change (24–26). The DALYs at each specific location were derived using the following formula:


$${\mathrm{DALY}}_{\mathrm{ax},px,\mathrm{ex}}=\overset{20}{\underset{i=1}{\sum }}\left(a_{i,x}*p_{x}*e_{i,x}\right)$$

where DALY a_x_, p_x_, e_x_ represented DALYs computed based on the dynamics of aging, population growth, and DALYs rate for year *x*. The a_i,x_ denoted the fraction of the population within age group i across the 20 age group in year x. The p_x_ signified the total population in year *x*. The e_i,x_ indicated DALYs rate for age group i in year *x*. The influence of each factor on the shift in DALYs from 1990 to 2019 was determined by assessing the impact of altering one factor while keeping the others constant.

### 2.5 Frontier analysis

The analytical methodology employed aimed to determine the minimum attainable burden of NDs based on the development status of countries or territories, as measured by SDI. The frontier represents the nations or regions at the forefront of performance, with the lowest burden of NDs relative to their SDI. The “effective difference” is calculated as the distance between the observed burden and the potentially achievable burden of disease for a country or territory, considering its specific SDI (27). This disparity could potentially be reduced or eliminated through the allocation and utilization of sociodemographic resources within the respective nation or region.

### 2.6 Slope and concentration indices of inequality analysis

For the inequality analysis, both the total DALYs and ASR-DALYs were extracted. Following the guidance of the World Health Organization, two standard indicators were applied to assess absolute and relative income-related disparities among countries (28). These indicators included the slope index of inequality (SII) and the concentration index (CI). The SII quantified the slope of the regression line relating the country-level ASR-DALYs for NDs to the weighted ranking of each country. Conversely, the CI was employed to assess the relative inequality in the burden of NDs experienced across countries. This was achieved by constructing a Lorenz concentration curve based on the cumulative distribution of DALYs and population. The CI represents the integrated area under the curve, varying in value between −1 and 1. A negative CI value indicates that the burden of NDs is more concentrated among populations in countries with lower SDI values (29).

## 3 Results

### 3.1 Global burden

**Figure 1:**
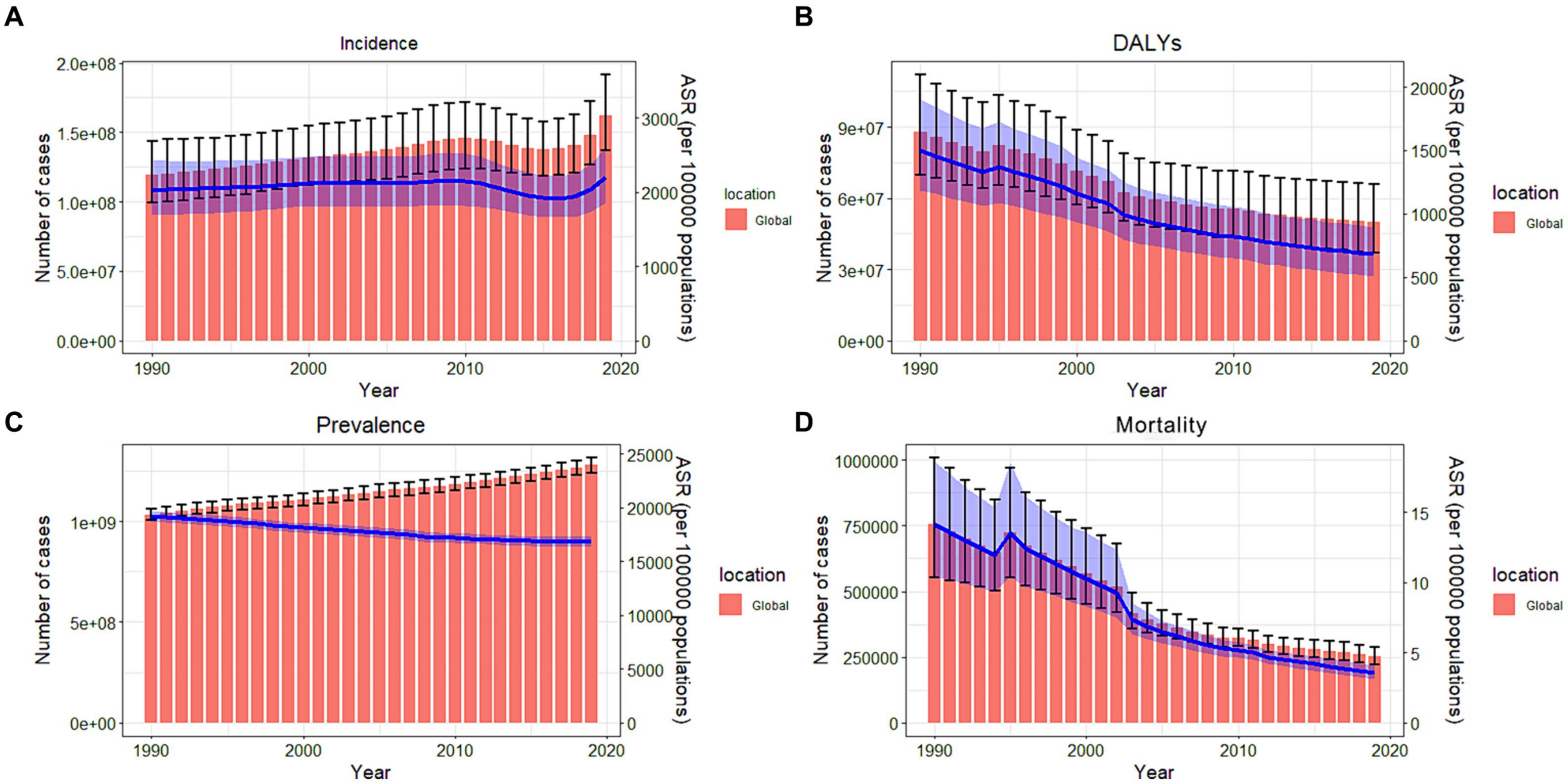
Bar and line charts for the global burden of nutritional deficiencies from 1990 to 2019. **(A)** Bar chart of the number–incidence and line chart of ASR–incidence; **(B)** Bar chart of the number–DALYs and line chart of ASR–DALYs; **(C)** Bar chart of the number–prevalence and line chart of ASR–prevalence; **(D)** Bar chart of the number–mortality and line chart of ASR–mortality. ASR, age-standardized rates; DALYs, disability-adjusted life years.

The incidence, DALYs, prevalence, and mortality of NDs globally, disaggregated by sex, age group, and region, are presented in terms of number, rate, and 95% uncertainty intervals (UIs). Additionally, the EAPCs and their 95% CIs are summarized in Table 1 and Supplementary Table S1. The trends in number and age-standardized rates from 1990 to 2019 are presented in Figure 1. According to GBD, the global incidence of NDs was 162,197,527.19 (95% UI: 136,653,963.59–191,299,880.24) in 2019. The ASIR of NDs was 2,207.71 per 100,000 individuals (95% UI: 1,863.04–2,604.67) in 2019, with an EAPC of 0.06 (95% CI: −11.8 to 13.51) from 1990 to 2019, indicating a modest upward trend. The number of DALYs was 49,775,123.92 (95% UI 36,889,949.52–65,839,421.79) in 2019, and the ASR-DALYs showed a significant decrease (EAPC-2.81, 95% CI: 14.32 to 10.25) from 1990 to 2019. The ASPR was 16,834.56 per 100,000 individuals (95% UI: 16,336.72–17,312.89) in 2019, whereas the EAPC was −0.13 (95% CI−11.95 to 13.29). In 2019, there was a significant reduction in the ASMR to 3.52 per 100,000 individuals (95% UI: 3.09–4.06), while the EAPC was −4.92 (−16.14 to 7.8).

**Table 1:** The number and ASR of incidents and DALYs for nutritional deficiency in 2019 and changing trends from 1990 to 2019.

	2019		1990–2019	2019		1990–2019
	Incidence cases	ASIR per 100,000 (95% UI)	EAPC of ASIR (95% CI)	DALYs cases	ASR-DALYs per 100,000 (95% UI)	EAPC of ASR-DALYs (95% CI)
Global	162,197,527.19 (136,653,963.59–191,299,880.24)	2,207.71 (1,863.04–2,604.67)	0.06 (−11.8 to 13.51)	49,775,123.92 (36,889,949.52–65,839,421.79)	680.12 (507.21–894.89)	−2.81 (−14.32 to 10.25)
Age (years)						
<5	61,336,085.27 (48,921,131.37–77,165,328.80)	9,253.49 (7,380.50–11,641.58)	−0.59 (−12.74 to 13.25)	16,004,379.25 (12,805,277.82–19,785,537.55)	2,414.51 (1,931.87–2,984.95)	−4.44 (−16.12 to 8.86)
5–9	7,318,804.66 (5,174,825.88–10,060,282.56)	1,117.88 (790.41–1,536.62)	−0.09 (−12.25 to 13.76)	6,569,570.67 (4,542,055.90–9,211,068.38)	1,003.44 (693.76–1,406.91)	−1.06 (−13.1 to 12.66)
10–14	8,166,559.93 (6,113,007.79–10,707,540.97)	1,271.68 (951.91–1,667.36)	0.20 (−11.97 to 14.05)	3,765,628.26 (2,551,977.86–5,350,961.72)	586.38 (397.39–833.24)	−0.43 (−12.52 to 13.34)
15–19	8,706,024.90 (6,800,766.70–11,261,649.25)	1,405.24 (1,097.71–1,817.74)	0.64 (−11.54 to 14.49)	2,552,170.79 (1,726,500.69–3,655,049.35)	411.95 (278.67–589.96)	−0.13 (−12.21 to 13.61)
20–24	8,687,982.95 (6,888,450.26–11,011,094.59)	1,447.65 (1,147.80–1,834.74)	0.85 (−11.28 to 14.65)	1,851,689.88 (1,251,334.15–2,619,724.72)	308.54 (208.51–436.52)	−0.89 (−12.82 to 12.66)
25–29	8,603,526.81 (6,875,676.83–10,906,198.90)	1,420.97 (1,135.59–1,801.28)	0.96 (−11.12 to 14.68)	1,831,764.85 (1,230,545.58–2,568,258.53)	302.54 (203.24–424.18)	−0.89 (−12.75 to 12.58)
30–34	8,481,904.29 (6,799,702.57–10,704,717.32)	1,409.58 (1,130.02–1,778.98)	1.11 (−10.89 to 14.73)	1,912,596.22 (1,293,594.93–2,698,019.37)	317.85 (214.98–448.38)	−0.78 (−12.56 to 12.59)
35–39	7,554,730.38 (6,019,939.06–9,453,514.54)	1,396.50 (1,112.79–1,747.49)	1.31 (−10.61 to 14.82)	2,127,198.36 (1,453,195.90–2,996,981.93)	393.21 (268.62–554.00)	−0.6 (−12.29 to 12.65)
40–44	7,043,554.77 (5,600,497.60–8,693,065.17)	1,427.43 (1,134.98–1,761.72)	1.67 (−10.15 to 15.05)	2,105,360.56 (1,447,835.26–3,005,432.26)	426.67 (293.41–609.07)	−0.52 (−12.09 to 12.56)
45–49	7,096,890.05 (5,622,700.81–8,723,859.59)	1,497.86 (1,186.72–1,841.25)	2.04 (−9.63 to 15.22)	1,941,721.57 (1,353,068.99–2,738,005.06)	409.82 (285.58–577.88)	−0.52 (−11.9 to 12.33)
50–54	6,765,503.16 (5,342,176.63–8,301,504.55)	1,548.82 (1,222.98–1,900.45)	2.19 (−9.28 to 15.11)	1,835,448.48 (1,277,555.21–2,532,792.82)	420.19 (292.47–579.83)	−0.64 (−11.79 to 11.92)
55–59	5,755,312.57 (4,550,976.09–7,058,040.40)	1,551.24 (1,226.64–1,902.37)	2.10 (−9.13 to 14.71)	1,705,806.70 (1,237,015.78–2,375,372.44)	459.77 (333.42–640.24)	−1.00 (−11.89 to 11.23)
60–64	4,930,269.58 (3,859,650.58–6,102,774.13)	1,577.51 (1,234.95–1,952.67)	2.11 (−8.88 to 14.44)	1,399,745.36 (1,015,010.61–1,868,496.72)	447.87 (324.77–597.85)	−1.20 (−11.84 to 10.73)
65–69	4,190,121.99 (3,254,324.81–5,265,031.25)	1,620.41 (1,258.52–2,036.10)	2.08 (−8.61 to 14.03)	1,268,238.51 (938,888.36–1,680,646.09)	490.46 (363.09–649.94)	−1.19 (−11.54 to 10.37)
70–74	3,071,339.09 (2,365,433.98–3,890,353.06)	1,641.66 (1,264.35–2,079.43)	2.15 (−8.15 to 13.62)	1,014,693.89 (779,860.07–1,312,955.89)	542.36 (416.84–701.79)	−1.07 (−11.05 to 10.04)
75–79	2,089,776.29 (1,594,316.93–2,662,661.54)	1,644.80 (1,254.84–2,095.70)	2.43 (−7.4 to 13.31)	787,704.57 (631,809.63–997,102.65)	619.98 (497.28–784.79)	−1.00 (−10.5 to 9.52)
80–84	1,381,786.91 (1,049,644.53–1,767,665.54)	1,636.76 (1,243.33–2,093.84)	2.66 (−6.49 to 12.7)	541,448.44 (445,406.16–664,397.12)	641.36 (527.59–786.99)	−0.81 (−9.65 to 8.9)
85–89	696,311.37 (525,454.77–896,520.84)	1,601.42 (1,208.47–2,061.87)	2.74 (−5.37 to 11.54)	345,108.31 (287,662.76–409,215.76)	793.70 (661.58–941.14)	−0.64 (−8.48 to 7.88)
90–94	252,005.88 (187,913.00–325,766.16)	1,494.91 (1,114.71–1,932.46)	2.89 (−3.71 to 9.95)	155,440.40 (131,212.36–178,155.09)	922.08 (778.36–1,056.82)	−0.14 (−6.55 to 6.72)
95+	69,036.32 (50,738.13–89,593.18)	1,446.33 (1,062.98–1,877.00)	3.32 (−1.39 to 8.27)	59,408.85 (47,860.93–66,962.46)	1,244.63 (1,002.70–1,402.88)	0.91 (−3.71 to 5.74)
Sex						
Male	88,861,045.53 (74,392,565.44–105,463,457.83)	2,382.12 (1,995.86–2,815.90)	0.22 (−10.96 to 12.82)	21,318,568.86 (16,030,317.36–27,817,401.49)	584.39 (441.45–763.20)	−3.14 (−13.95 to 9.02)
Female	73,336,481.66 (62,109,682.53–86,022,373.68)	2,034.41 (1,726.61–2,385.48)	−0.14 (−11.26 to 12.38)	28,456,555.06 (21,132,185.90–37,721,020.92)	777.92 (578.51–1,028.93)	−2.54 (−13.39 to 9.68)
Cause						
Vitamin A deficiency	489,662,708.61 (469,006,373.61–512,234,291.26)	6,328.46 (6,061.50–6,620.18)	−2.79(−14.28 to 10.23)	1,177,507.28 (805,056.04–1,636,582.37)	15.22 (10.40–21.15)	−2.34(−13.91 to 10.79)
Protein–energy malnutrition	154,086,018.76 (128,445,222.55–183,279,047.29)	1,991.43 (1,660.04–2,368.72)	0.08 (−11.78 to 13.53)	15,256,524.16 (12,565,113.57–18,327,802.94)	197.18 (162.39–236.87)	−5.13(−16.37 to 7.61)
Iodine deficiency	8,111,508.43 (6,500,143.19–9,966,056.95)	104.83 (84.01–128.80)	−0.35 (−12.18 to 13.07)	2,438,598.59 (1,372,657.05–4,238,613.26)	31.52 (17.74–54.78)	−0.85(−12.61 to 12.49)
Dietary iron deficiency	–	–	–	28,534,680.33 (19,127,591.04–41,139,284.42)	368.79 (247.21–531.69)	−0.36(−12.16 to 13.02)
Other nutritional deficiencies	–	–	–	2,367,813.56 (1,879,521.69–2,950,808.96)	30.60 (24.29–38.14)	−3.95(−15.32 to 8.94)
SDI						
High	10,845,727.58 (8,773,025.28–13,098,261.68)	1,062.62 (870.67–1,295.90)	0.54 (−9.37 to 11.54)	1,456,033.74 (1,049,926.14–1,973,129.65)	130.80 (90.96–180.72)	−0.81 (−10.61 to 10.07)
High–middle	22,294,078.59 (18,224,647.33–27,126,082.63)	1,879.06 (1,540.14–2,291.77)	1.36 (−8.87 to 12.73)	3,777,225.09 (2,628,068.31–5,243,541.89)	275.47 (189.75–384.60)	−2.1 (−12.07 to 9)
Middle	43,941,881.53 (37,056,079.97–52,709,211.06)	2,223.00 (1,846.85–2,643.85)	0.77 (−9.88 to 12.69)	10,018,417.98 (7,136,818.09–13,547,618.69)	445.72 (320.11–601.15)	−2.35 (−12.76 to 9.3)
Low–middle	38,142,885.45 (33,348,332.11–44,541,075.57)	2,707.55 (2,289.16–3,191.73)	−0.47 (−10.66 to 10.88)	16,197,717.42 (11,889,739.54–21,765,779.08)	946.24 (697.13–1,264.18)	−3.98 (−13.92 to 7.11)
Low	22,852,411.46 (20,865,621.77–25,426,216.97)	2,153.30 (1,866.44–2,483.55)	−0.15 (−9.84 to 10.57)	18,299,216.99 (14,184,879.76–23,321,623.40)	1,412.48 (1,090.05–1,802.47)	−2.86 (−12.41 to 7.73)
GBD region						
Central Asia	802,197.56 (674,819.35–960,613.05)	859.57 (720.26–1,035.03)	−0.14 (−7.47 to 7.77)	466,712.94 (310,558.78–663,200.58)	495.98 (331.08–705.73)	−1.17 (−8.39 to 6.63)
East Asia	29,341,667.20 (23,187,620.65–36,344,214.77)	2,051.36 (1,628.87–2,558.92)	0.81 (−9.51 to 12.31)	2,736,622.23 (1,906,356.40–3,833,471.52)	178.06 (124.61–245.82)	−5.57 (−15.25 to 5.21)
South Asia	66,381,377.25 (55,381,876.92–78,770,018.40)	3,811.20 (3,186.51–4,524.80)	−0.39 (−10.73 to 11.14)	20,946,036.76 (14,876,928.43–28,637,409.92)	1,208.99 (863.74–1,650.24)	−3.26 (−13.27 to 7.9)
Southeast Asia	16,813,153.70 (14,213,232.76–19,563,548.15)	2,655.93 (2,253.18–3,099.13)	−0.11 (−9.48 to 10.22)	3,162,731.69 (2,361,354.17–4,261,032.49)	509.59 (386.17–678.62)	−2.85 (−11.92 to 7.17)
High-income Asia Pacific	1,323,670.16 (1,075,415.05–1,613,425.55)	814.85 (688.18–970.00)	−0.16 (−8.27 to 8.66)	317,743.13 (215,859.57–455,272.84)	160.83 (106.93–234.03)	−2.15 (−10.17 to 6.59)
North Africa and the Middle East	9,359,302.78 (8,040,842.40–10,846,678.65)	1,567.26 (1,349.63–1,818.62)	0.02 (−9.21 to 10.19)	2,136,442.82 (1,525,523.77–2,983,496.56)	357.26 (256.56–495.56)	−2.37 (−11.36 to 7.54)
Central Sub-Saharan Africa	2,746,499.44 (2,373,992.81–3,206,242.16)	1,590.10 (1,384.00–1,831.11)	−0.56 (−8.14 to 7.65)	1,739,853.22 (1,284,271.95–2,316,849.17)	1,197.25 (893.38–1,577.78)	−3.23 (−10.56 to 4.69)
Eastern Sub-Saharan Africa	7,142,597.28 (6,072,121.87–8,380,018.96)	1,407.02 (1,222.45–1,610.12)	−0.69 (−9.44 to 8.9)	6,298,676.09 (4,988,235.71–7,823,056.37)	1,340.47 (1,073.05–1,633.14)	−3.81 (−12.27 to 5.47)
Southern Sub-Saharan Africa	842,428.18 (716,190.01–992,424.69)	1,077.73 (916.13–1,266.98)	−0.58 (−7.69 to 7.07)	632,595.19 (498,189.36–787,207.99)	820.52 (650.92–1,014.44)	−1.21 (−8.27 to 6.39)
Western Sub-Saharan Africa	9,412,428.44 (8,157,715.05–11,017,349.35)	1,579.86 (1,377.17–1,814.37)	−0.02 (−8.92 to 9.76)	7,125,741.57 (5,449,404.38–9,185,620.89)	1,187.83 (906.35–1,534.48)	−1.58 (−10.34 to 8.05)
Andean Latin America	377,625.14 (332,460.73–431,579.16)	606.33 (534.40–692.12)	−0.6 (−7.36 to 6.66)	240,934.88 (179,774.48–317,266.41)	388.51 (290.70–509.05)	−4.8 (−11.31 to 2.19)
Central Latin America	2,975,635.59 (2,483,002.11–3,527,053.71)	1,225.11 (1,025.85–1,449.23)	−0.57 (−8.85 to 8.46)	768,753.44 (612,601.74–963,122.99)	326.12 (260.72–406.18)	−3.7 (−11.71 to 5.04)
Southern Latin America	605,049.20 (501,196.63–727,121.43)	910.67 (761.15–1,090.04)	0.65 (−6.37 to 8.2)	132,057.23 (97,090.63–180,802.14)	201.20 (144.31–279.53)	−2.37 (−9.17 to 4.95)
Tropical Latin America	1,439,938.29 (1,206,774.99–1,712,146.47)	698.00 (591.64–819.69)	−0.96 (−9.08 to 7.89)	971,907.86 (711,188.31–1,356,677.96)	455.67 (335.18–627.47)	−3.36 (−11.29 to 5.27)
High-income North America	3,072,006.01 (2,418,972.01–3,776,252.74)	792.51 (628.56–977.80)	0.35 (−8.49 to 10.04)	443,235.15 (322,521.87–601,267.28)	108.42 (76.49–152.22)	0.49 (−8.39 to 10.22)
Caribbean	422,723.13 (356,806.51–499,163.96)	949.86 (801.63–1,124.92)	−0.72 (−7.3 to 6.32)	292,017.48 (220,700.44–380,301.11)	664.18 (499.77–867.81)	−2.21 (−8.7 to 4.73)
Australasia	166,503.41 (138,737.07–199,068.10)	613.40 (522.66–723.39)	1.09 (−4.99 to 7.55)	25,808.97 (17,291.93–36,906.28)	90.08 (57.41–134.10)	−0.76 (−6.75 to 5.62)
Oceania	267,590.02 (214,799.43–331,684.04)	1,675.45 (1,394.53–2,012.58)	0.34 (−4.84 to 5.79)	96,886.57 (68,687.64–133,223.18)	704.11 (506.66–957.36)	−0.07 (−5.13 to 5.26)
Central Europe	944,061.56 (754,099.79–1,170,502.58)	1,053.97 (825.37–1,340.30)	0.39 (−7.25 to 8.66)	193,280.70 (129,244.85–277,703.47)	188.67 (123.68–275.77)	−1.71 (−9.26 to 6.48)
Eastern Europe	1,539,084.02 (1,223,373.03–1,896,298.72)	1,007.71 (803.38–1,261.20)	0.17 (−8.05 to 9.11)	379,716.35 (258,871.17–546,433.49)	163.54 (110.35–234.02)	−1.75 (−10 to 7.26)
Western Europe	6,221,988.85 (5,080,799.40–7,541,775.94)	1,364.45 (1,117.74–1,673.15)	0.64 (−8.46 to 10.65)	667,369.65 (470,188.01–896,058.65)	133.98 (91.14–184.81)	−0.3 (−9.35 to 9.64)

**Figure 2:**
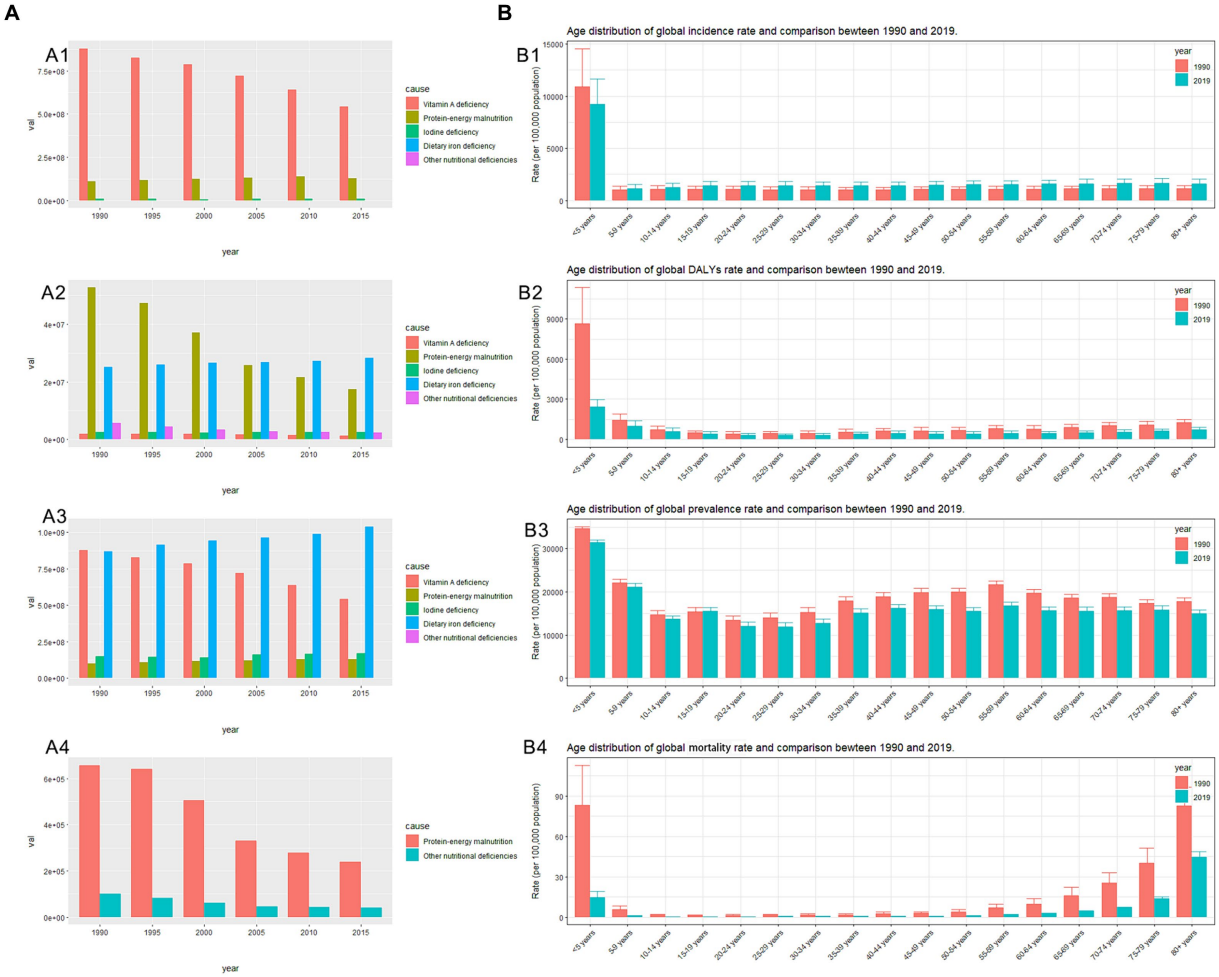
**(A)** Cause distribution of nutritional deficiencies and comparison between 1990 and 2019. **(A1)** Number of global incidences; **(A2)** Number of global DALYs; **(A3)** Number of global prevalence; **(A4)** Number of global mortality. **(B)** Age distribution of nutritional deficiencies and comparison between 1990 and 2019. **(B1)** Global ASR–incidence; **(B2)** Global ASR–DALYs; **(B3)** Global ASR–prevalence; **(B4)** Global ASR–mortality. ASR, age-standardized rates; DALYs, disability-adjusted life years.

NDs, particularly those caused by vitamin A deficiency, exhibited the highest incidence (489,662,708.61, 95% UI: 469,006,373.61–512,234,291.26) and the highest ASIR (6,328.46, 95% UI: 6,061.50–6,620.18) (Table 1). However, the number of vitamin A deficiency cases showed a gradual reduction from 1990 to 2019 (Figure 2A1). Among the five factors contributing to DALYs from NDs, protein–energy malnutrition and dietary iron deficiency are notable. The number and ASR-DALYs of protein–energy malnutrition are decreasing (Table 1; Figure 2A2), whereas dietary iron deficiency remains a concern. DALYs due to dietary iron deficiency exhibited an increasing trend (Figure 2A2), with the ASR-DALYs associated with it ranking highest and showing minimal variation (Table 1). In addition, dietary iron deficiency ranks highest in prevalence in both terms of numbers and ASPR, with a slight decrease noted in ASPR (Figure 2A3; Supplementary Table S1). In the 2019 GBD database, only two causes—protein–energy malnutrition and other NDs—were represented, with protein–energy malnutrition resulting in a significantly higher number of mortality and ASMR than the other NDs (Supplementary Table S1; Figure 2A4).

Despite a remarkable downward trend, children under the age of 5 years continue to face the highest risk of NDs. This is evidenced by their highest incidence, prevalence, DALYs, and mortality (Table 1; Supplementary Table S1) and the highest ASIR, ASR-DALYs, and ASPR across all age groups (Figure 2B). Among children aged under <5 years, females had a higher incidence and prevalence, while males exhibited a higher number of DALYs and mortality (Supplementary Figure S1). In this age group, while the incidence and prevalence of protein–energy malnutrition were relatively low, the number of DALYs and mortality were notably high (Supplementary Figure S1). Among older adults (aged ≥65), there is a progressive increase in the ASMR with advancing age, primarily attributed to protein–energy malnutrition (Supplementary Figure S4).

**Figure 3:**
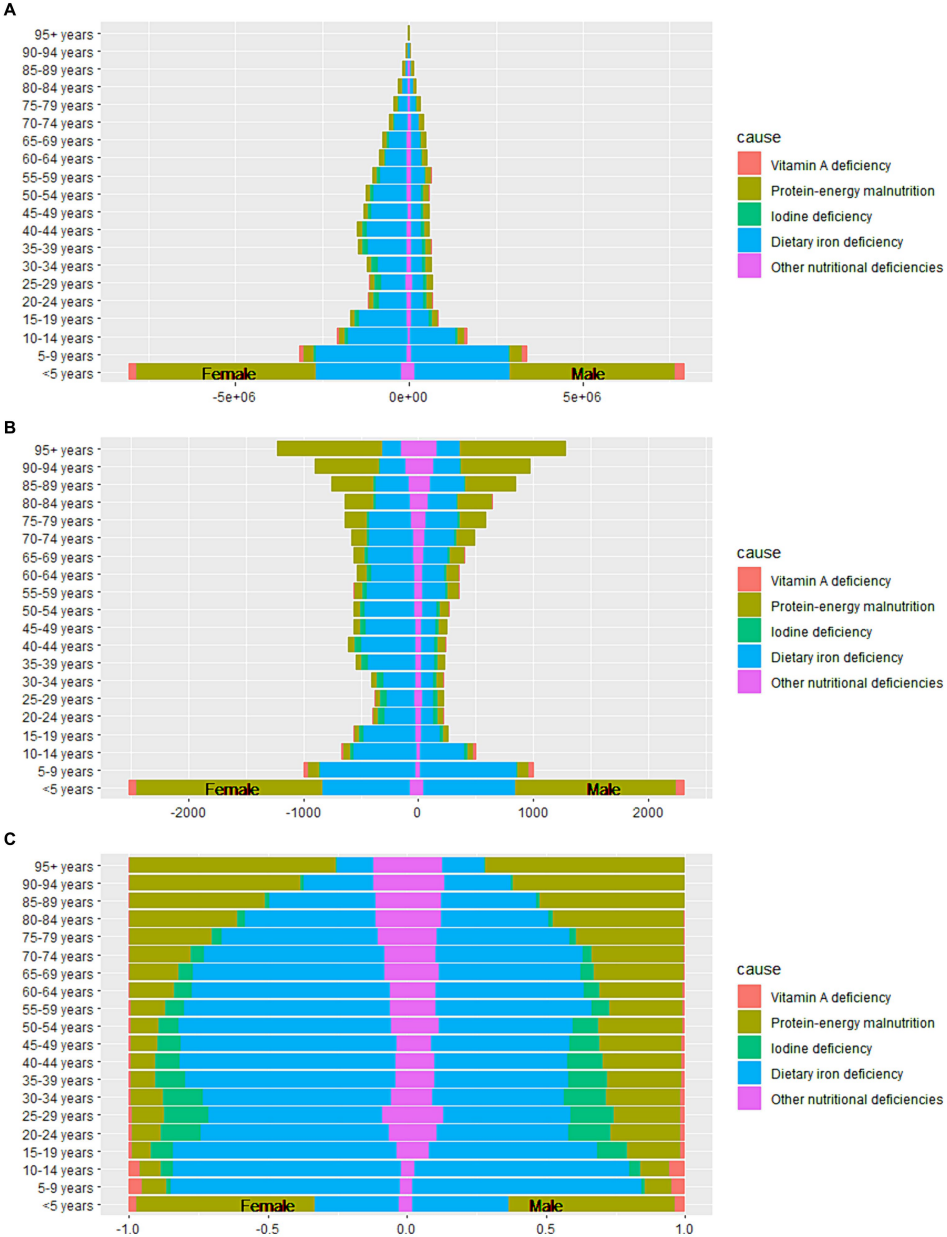
Sex-differentiated age-specific distribution of causes for nutritional deficiencies. **(A)** Number of DALYs; **(B)** ASR-DALYs; **(C)** Proportion of DALYs. DALYs, disability-adjusted life years; ASR, age-standardized rates.

The ASIR of NDs in males is higher than that in females. Sex differences in ASR-DALYs, ASPR, and ASMR reveal lower rates in males than in females (Table 1; Supplementary Table S1). From the age of 10 years to under the age of 80 years, females experience a higher prevalence of dietary iron deficiency and corresponding ASR-DALYs than do males (Figure 3). Across all age groups, the ASIR of protein–energy malnutrition was consistently lower in females than in males (Supplementary Figure S2). In each age group, the females also exhibit higher prevalence rates of iodine deficiency than did males (Supplementary Figure S3).

**Figure 4:**
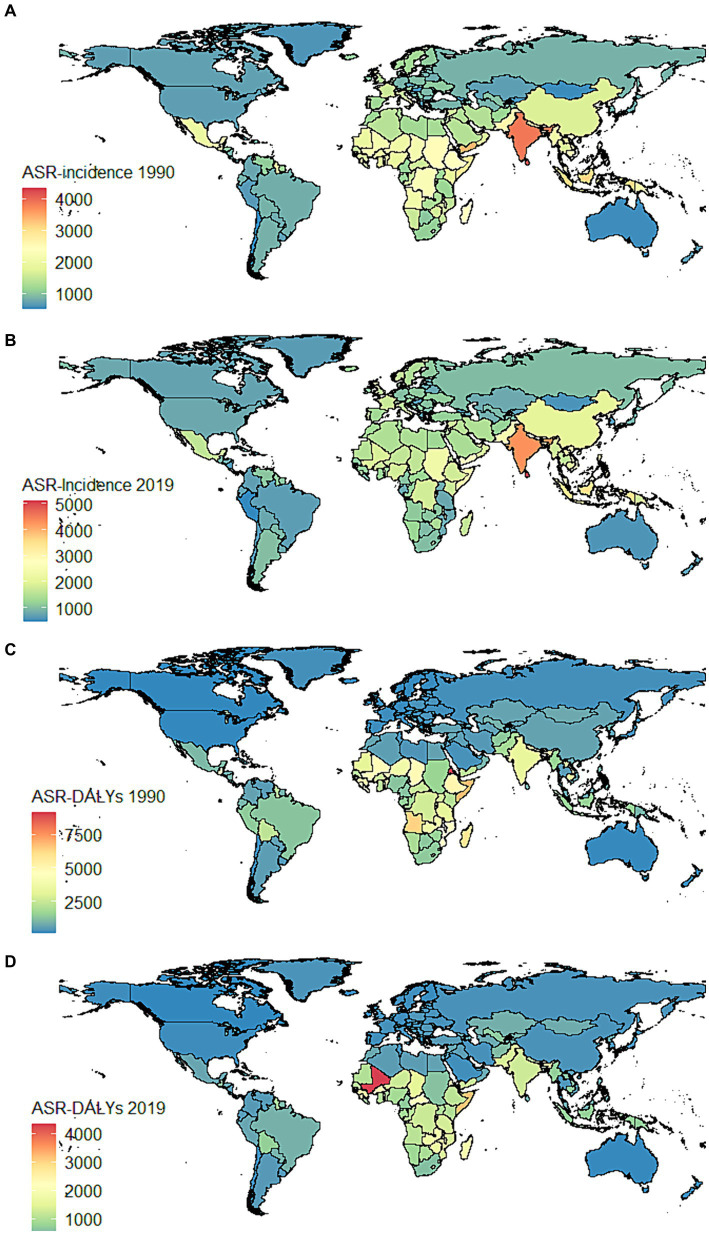
Global distribution maps for the burden of nutritional deficiencies in 204 countries and territories in 1990 and 2019. **(A)** ASR–incidence in 1990; **(B)** ASR–incidence in 2019; **(C)** ASR–DALYs in 1990; **(D)** ASR–DALYs in 2019. ASR, age-standardized rates; DALYs, disability-adjusted life years.

It has been observed that regions with high and medium-high SDI have lower ASIR in NDs, yet the EAPC of ASIR has increased in these regions (Table 1). South Asia had the highest number of cases and the highest ASIR among all GBD regions (Table 1). Particularly in India, the ASIR did not show significant improvement from 1990 to 2019 (Figures 4A,B). In addition, the ASR-DALYs increased in high-income North America within the GBD region (EAPC 0.49, 95% CI: −8.39 to 10.22) (Table 1), and ASR-DALYs increased notably in the country of Mali (Figures 4C,D).

Over the past three decades, there has been an increase in the ASPR in regions with low SDI (Table 1). The ASPR has increased in South Asia, particularly India (Supplementary Figures S5A,B). The high SDI region exhibited a slight upward trend in ASMR, with an EAPC of 0.71 (95% CI: −9.49 to 12.05). In addition, a significant escalation in ASMR was detected in Mali and Somalia (Supplementary Figures S5A,B). Countries with large populations, such as China and India, have reported high incidence, DALYs, prevalence, and mortality (Supplementary Figures S6, S7).

### 3.2 Health inequality

**Figure 5:**
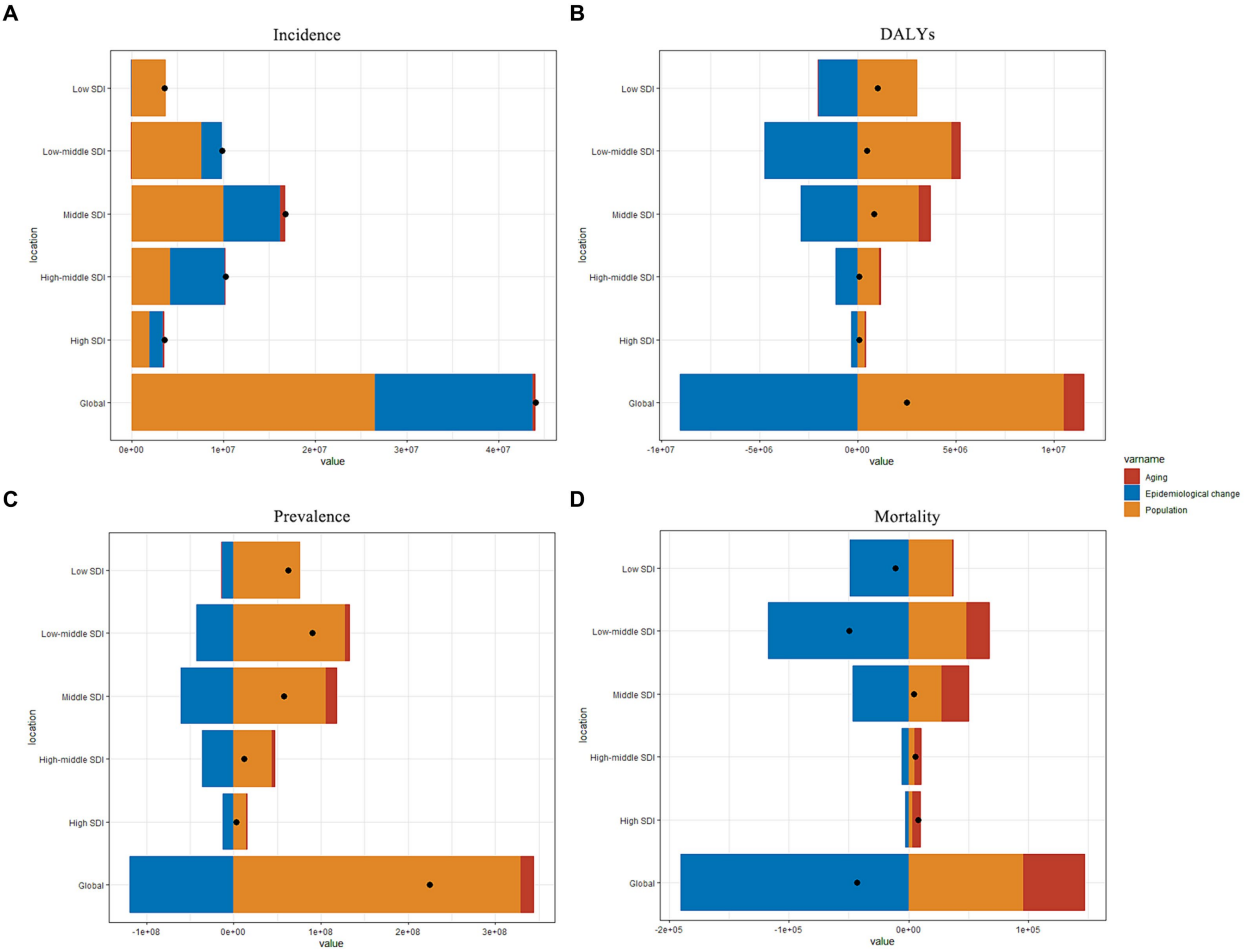
Changes in nutritional deficiencies according to population-level determinants of population growth, aging, and epidemiological change from 1990 to 2019 at the global level and by SDI quintile (The black dot represents the overall value of change contributed by all three components). **(A)** Incidence rate; **(B)** Prevalence rate; **(C)** DALYs rate; **(D)** Mortality rate. SDI, Sociodemographic Index; DALYs, disability-adjusted life years.

Decomposition analysis was conducted to assess how factors such as aging, population growth, and epidemiologic changes impact the epidemiology of NDs (Figure 5). Between 1990 and 2019, population growth followed by epidemiological changes emerged as the primary drivers of increased incidence globally (Figure 5A). The contribution of aging to overall incidence decreased in regions with low-SDI regions but was more pronounced in middle-SDI regions. Population growth had a contrasting effect on the burden of DALYs and prevalence, while the impact of aging showed a decreasing trend (Figures 5B,C). In terms of DALYs, the most significant increase was observed in the low-SDI region, while the low–middle SDI region experienced the highest increase in prevalence. Overall, there was a decline in global mortality, particularly notable in the low–middle SDI region, followed by the low–middle SDI region. In addition, middle-SDI, middle–high SDI, and high-SDI regions exhibited varying degrees of increase in mortality (Figure 5D). The contribution of aging increased as SDI levels transitioned from low to high.

**Figure 6:**
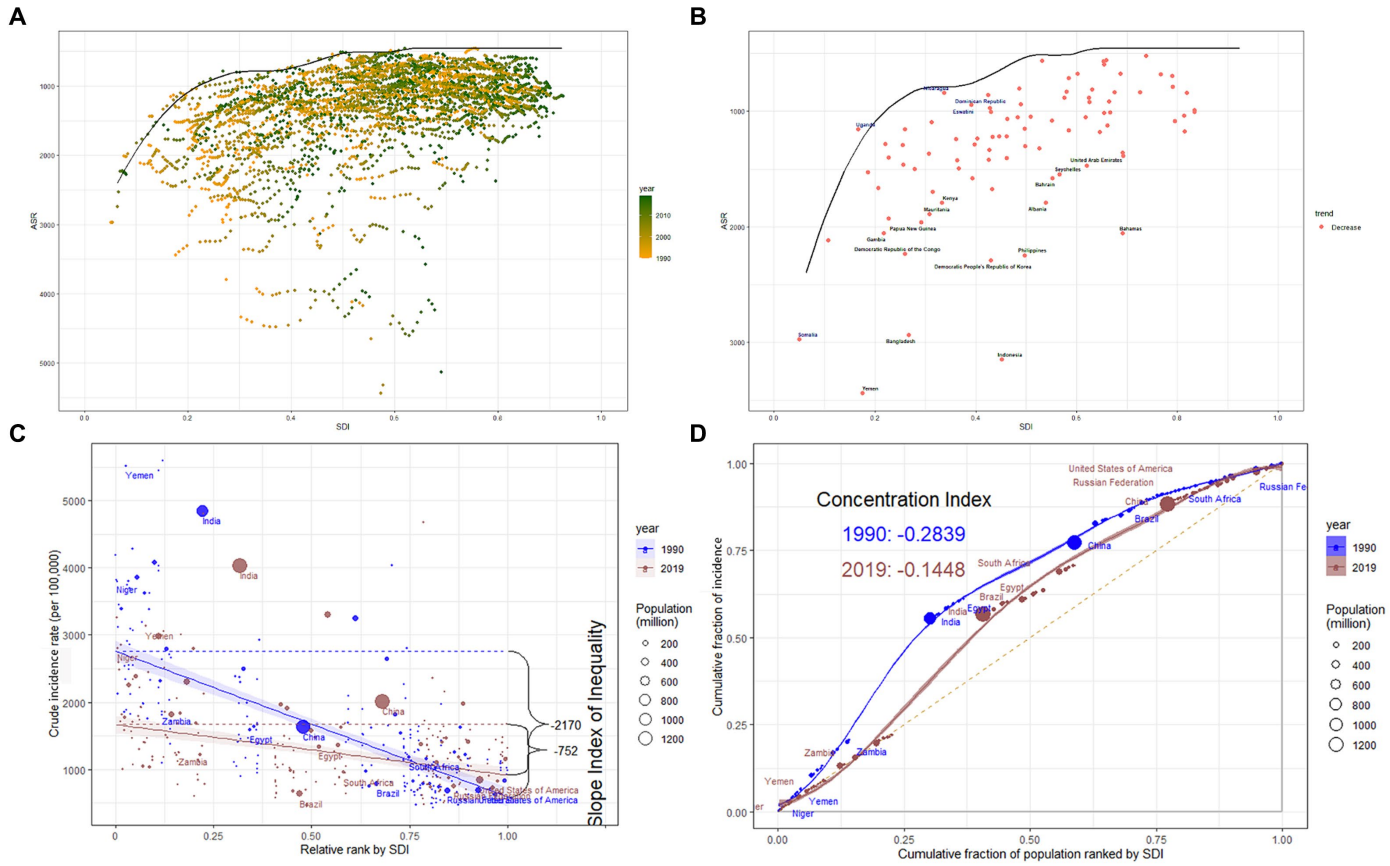
**(A)** Frontier analysis based on SDI and ASR-incidence of nutritional deficiencies from 1990 to 2019. The color scale represents the years from 1990 depicted in orange to 2019 depicted in green. The frontier is delineated in a solid black color. **(B)** Frontier analysis based on SDI and ASR-incidence of nutritional deficiencies trend between 1990 and 2019. The frontier is delineated in solid black color; countries and territories are represented as dots. The top 15 countries with the largest effective difference (largest ASR-incidence gap from the frontier) are labeled in black; the top 5 countries with the lowest effective difference with low SDI (<0.5) are labeled in blue. The red dots indicate a decrease in ASR incidence from 1990 to 2019. **(C)** Slope indices inequality for incidence of nutritional deficiencies in 1990 and 2019 (the numbers adjacent to the brackets indicate the slopes). **(D)** Concentration indices for incidence of nutritional deficiencies in 1990 and 2019 (Each country or region is represented by a solid dot, with larger dots indicating a higher population). SDI, Sociodemographic Index; ASR, age-standardized rates.

A frontier analysis was conducted utilizing data from 1990 to 2019, focusing on ASIR, ASR-DALYs, ASPR, ASMR, and SDI to enhance understanding of potential improvements in NDs. Our findings revealed that the effective difference (EF) for a specific SDI decreased as SDI increased, resulting in reduced variance. After the SDI surpasses 0.6, the frontier of ASIR tends to stabilize (Figure 6A). The frontiers for ASR-DALYs, ASPR, and ASMR reach stability at SDI thresholds of 0.35, 0.8, and 0.3, respectively (Supplementary Figures S8A, S9A, S10A). According to ASIR, Somalia, Uganda, Nicaragua, Dominican Republic, and Eswatini were identified as frontier countries with SDI < 0.5 (Figure 6B). The map of the frontier is clearly defined by a solid black line. Within this boundary, individual countries and territories are graphically represented as dots. Among countries with a low SDI of less than 0.5, the top 15 countries exhibiting the largest effective difference, as measured by the gap between their ASR–incidence and the global frontier, are annotated in black. Conversely, the top 5 countries with the smallest effective difference are identified in blue (Supplementary Figures S8B, S9B, S10B).

In 1990 and 2019, the SII (per 100,000 population) for incidence were −2170 and −752 (Figure 6C), for DALYs were −4301 and −1109 (Supplementary Figure S8C), for prevalence were −19926 and −18110 (Supplementary Figure S9C), and for mortality were −45.5 and −6.7 (Supplementary Figure S10C), indicating a negative correlation with SDI index. The marked reductions indicate a decrease in the disparity of the age-standardized burden of NDs between higher SDI and lower SDI nations over the observed period, except for ASPR.

The concentration curves and concentration index of ASIR, ASR-DALYs, ASPR, and ASMR are presented in Figure 6D and Supplementary Figures S8D, S9D, S10D, respectively. The concentration curves were above the equality line from 1990 to 2019, suggesting that the burden of NDs was more endemic in lower SDI regions. Between 1990 and 2019, there has been a consistent downward trend in the concentration index for incidence, DALYs, and mortality associated with the specified condition. Although the inequality in the burden of NDs has decreased, regional disparities still persist. This suggests that while economic inequalities have lessened in certain areas, global disparities in the burden of NDs continue to be a significant challenge.

## 4 Discussion

The global landscape from 1990 to 2019 exhibited a notable decline in both ARS-DALYs and ASMR of NDs; however, there was a slight increase in ASIR, indicating that the world has not yet fully escaped the impact of NDs. In particular, the disease burden among children under the age of 5 years has significantly improved. Nonetheless, given that this group remains at a heightened risk for NDs, continued effort to enhance their health status is imperative. The ASMR among individuals over the age of 65 years was notably high, with protein malnutrition identified as a primary factor, necessitating targeted intervention strategies to address this issue. Although male individuals experienced higher incidence and prevalence rates, female individuals exhibited higher levels of ARS-DALYs and ASMR. There has been an improvement in health equity regarding NDs, yet significant disparities persist. Mortality, incidence, and DALYs are trending downward in low and lower-middle SDI regions, yet these areas continue to bear a heavy disease burden. It is noteworthy that ARS-DALYs and ASMR are on the rise in high-SDI areas.

The global reduction in NDs can be attributed to the implementation of nutrition-sensitive agricultural strategies, representing a departure from the conventional focus on staple crops such as grains and oilseeds. This innovative approach promotes the diversification of food inputs by bolstering the cultivation of a wider spectrum of crops and the augmentation of aquaculture and livestock farming, including dairy farming activities. As a result, there has been an increase in the variety of nutritious food production and an improvement in the accessibility of these healthful foods for households, thus enhancing food security (30). Over the past 30 years, there has been ongoing advancement in the global social economy. Enhanced economic growth is correlated with increased availability of nourishing dietary options and improved medical interventions, resulting in better nutritional wellbeing. Empirical evidence has substantiated the correlation between socioeconomic factors and various diseases (31). Due to the disparate economic development across regions, a significant impediment for many households is the prohibitive cost of nutritious food items, which hampers their ability to procure a healthy diet. Recent estimates suggest that a staggering three billion individuals globally lack the financial means to afford a healthy eating regimen (32). The “2030 Agenda for Sustainable Development” includes 17 Sustainable Development Goals (SDGs), with SDG 2 focusing on Zero Hunger. By 2030, SDG 2 aims to end all forms of malnutrition, including achieving, by 2025, the internationally agreed targets for reducing stunting and wasting in children under 5 years of age and addressing the nutritional needs of adolescent girls, pregnant and lactating women, and older individuals (33). Achieving SDG 2 presents a daunting challenge.

NDs among children pose a substantial threat to societal and national wellbeing. It impairs health, affects education, and hinders individual development in the long run, while exerting a negative impact on human capital and economic growth (34). Consequently, it is crucial to refine strategies for addressing NDs in children; measures such as improving dietary quality, incorporating a greater variety of nutrient-rich foods, providing essential micronutrient supplements, curtailing the consumption of unhealthy items, and promoting physical activity are advocated. Active utilization of nutritional interventions in school-based environments, family and community settings, social protection frameworks, and technological platforms should be pursued (35). Concurrently, scholarly research has underscored the correlation between maternal malnutrition and the elevated risk of child malnutrition, thereby leading to potential health issues in children (36, 37). Accordingly, a persistent focus on the nutritional status of women of childbearing age is imperative.

A salient trend that warrants attention is the rapid growth of the older adult population. Forecasts from the United Nations suggest that by 2050, the number of individuals aged 65 years or above will double the number of children under the age of 5 years and will exceed the count of individuals aged 15–24 years. It is projected that improvement in survival rate will contribute to an approximate 5-year increase in life expectancy at birth globally, which was 72.6 years in 2019 (38). Among the elderly population, the incidence and burden of NDs escalate with advancing age. NDs, particularly protein–energy malnutrition, pose a significant threat to older adults and are linked to reduced muscle mass and function (39). This is associated with a spectrum of health concerns, including impaired wound healing, increased susceptibility to infections, anemia, and delayed recovery (40). Despite this, significant challenges persist in the comprehension, recognition, and management of malnutrition among older adults (41). Only a minority of high-income countries, including Australia, Singapore, and New Zealand, have developed nutritional guidelines or recommendations specifically tailored to older adults (42). However, the importance of addressing NDs in this population has not been adequately highlighted.

Dietary iron deficiency is the predominant contributing factor to the ASPR and ASR-DALYs of NDs. It particularly affects children, adolescents, and women, residing in low-and middle-income regions (43). The WHO advocates for the adoption of population-scale interventions to prevent dietary iron deficiencies. These interventions include the centralized enrichment of staple foods and seasonings with iron, home-based enrichment of infant complementary foods with iron and additional micronutrients, and the provision of daily or periodic iron supplements (44). In areas where dietary iron deficiency contributes to the prevalence of anemia, the WHO recommends a public health measure involving annual iron supplementation for a duration of 3 months for all children aged 6 months and older (45). The implementation of a comprehensive range of intervention strategies is essential to reduce the burden of disease caused by dietary iron deficiency.

Despite a global decline in the prevalence of NDs, certain countries or regions continue to face significant challenges. For example, in South Asia, there are notably high ASIR, ASR-DALYs, ASPR, and ASMR. In addition, Mali is currently grappling with heightened concerns stemming from food insecurity and armed conflicts, which have contributed to an increase in both ASR-DALYs and ASMR. In the coming years, it is imperative to enhance interregional collaboration to systematically disseminate medical advancement and promote health awareness in these countries or regions, with the aim of enhancing their healthcare capabilities (8).

The quality of data used in the GBD study varies across different countries, potentially leading to biases in estimates of disease burden. In low-and middle-income regions, where health data infrastructure may be less robust, the data may be incomplete (46). The statistical models utilized in the Global Burden of Disease (GBD) study, although sophisticated, rely on assumptions that may not adequately capture the unique characteristics of each population due to the heterogeneity of epidemiological landscapes among individual countries (3). Furthermore, the GBD 2019 focused solely on protein–energy malnutrition, dietary iron deficiency, vitamin A deficiency, and iodine deficiency. However, it lacked sufficient data to comprehensively evaluate other NDs, such as vitamin C and folate (10). Some data, including the incidence of dietary iron deficiency, were not included in the GBD 2019 study. Future research endeavors should aim to integrate supplementary metrics, such as the Health Assessment Questionnaire (HAQ), the Human Development Index (HDI), or a composite of diverse databases, to enhance the precision of evaluating disease burden and assessing health status.

## 5 Conclusion

Since the 1990s, extensive initiatives have led to a decrease in the global burden of NDs. However, the burden of NDs among children and the elderly remains high, and significant health inequalities persist across regions, reflecting variations in social development. Consequently, further studies should focus on the nutritional wellbeing of these vulnerable populations. In addition, international support should be increased for low-and low–middle SDI countries to reduce these disparities. These findings offer critical insights for the development of global health strategies with the aim of mitigating NDs, potentially guiding policymakers in the deployment of interventions that are both effective and economically viable.

## Data Availability

The datasets presented in this study can be found in online repositories. The names of the repository/repositories and accession number(s) can be found at: http://ghdx.healthdata.org/ihmedata.
