# Supplementary material for: Global burden and health inequality of nutritional deficiencies from 1990 to 2019
Source: Front Nutr. 2024 Sep 25;11:1470713. doi: 10.3389/fnut.2024.1470713 (PMC11461340; doi:10.3389/fnut.2024.1470713)
Supplement: Supplementary file 1 [file Data_Sheet_1.docx]

Supplementary Material

# Supplementary Figures and Tables

## Supplementary Figures


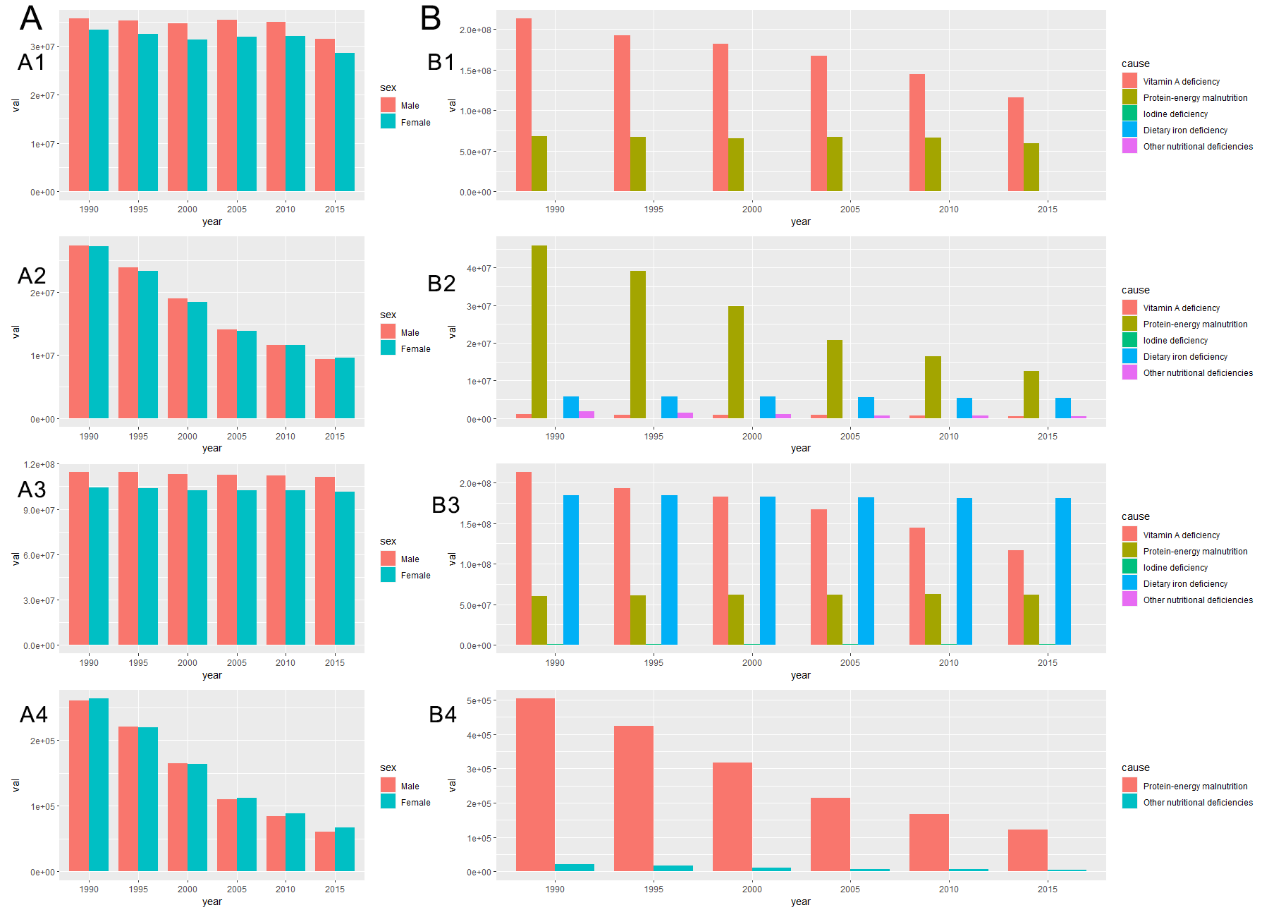


**Supplementary** **Figure 1.** (A) Sex distribution of nutritional deficiencies and comparison between 1990 and 2019 among child under the age of five. (A1) Number of global incidences; (A2) Number of global DALYs; (A3) Number of global prevalence; (A4) Number of global mortality. (B) Cause distribution of nutritional deficiencies and comparison between 1990 and 2019. (B1) Number of global incidences; (B2) Number of global DALYs; (B3) Number of global prevalence; (B4) Number of global mortality. DALYs: disability-adjusted life years.

**
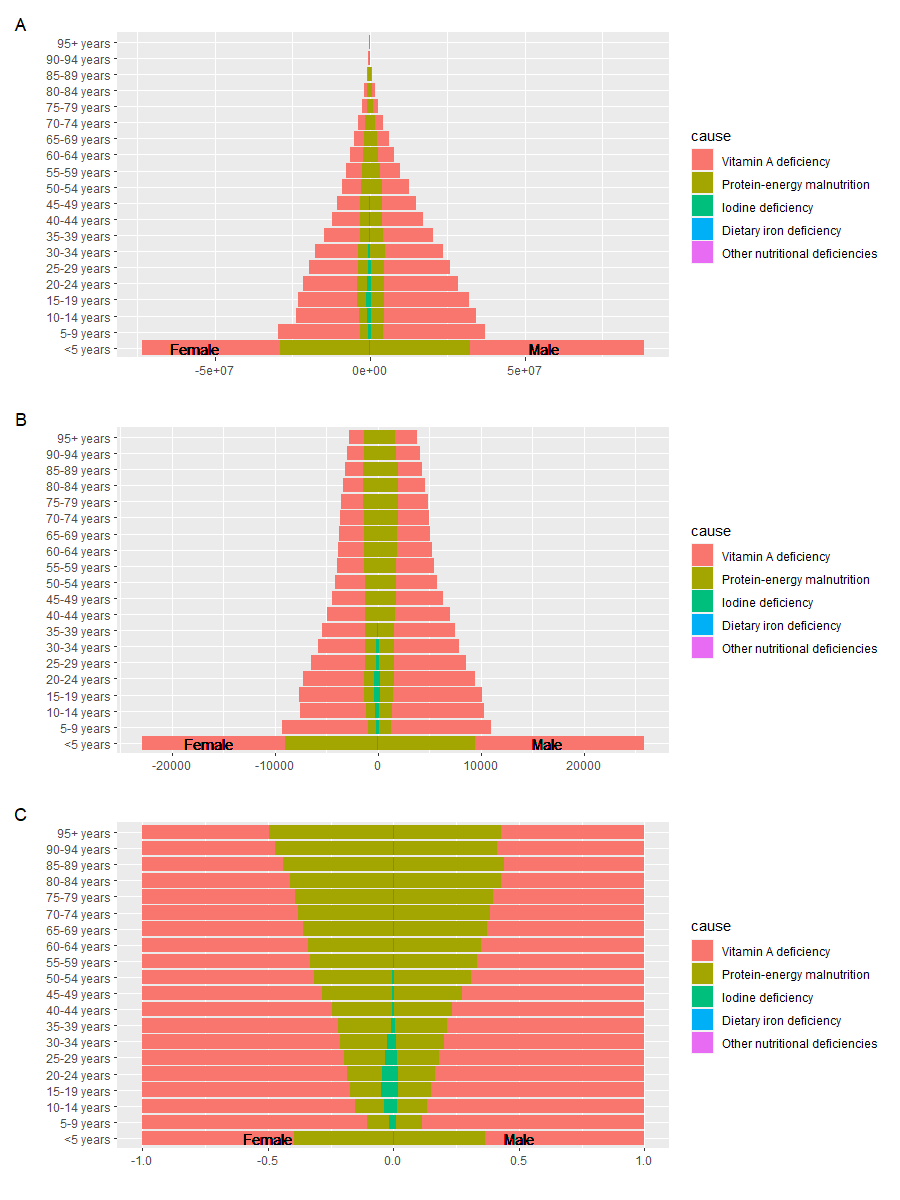
**

**Supplementary Figure 2.** Sex-differentiated Age-specific distribution of Causes for nutritional deficiencies. (A) Number of incidences; (B) ASR- incidence; (C) Proportion of incidence. ASR: age-standardized rates.

**
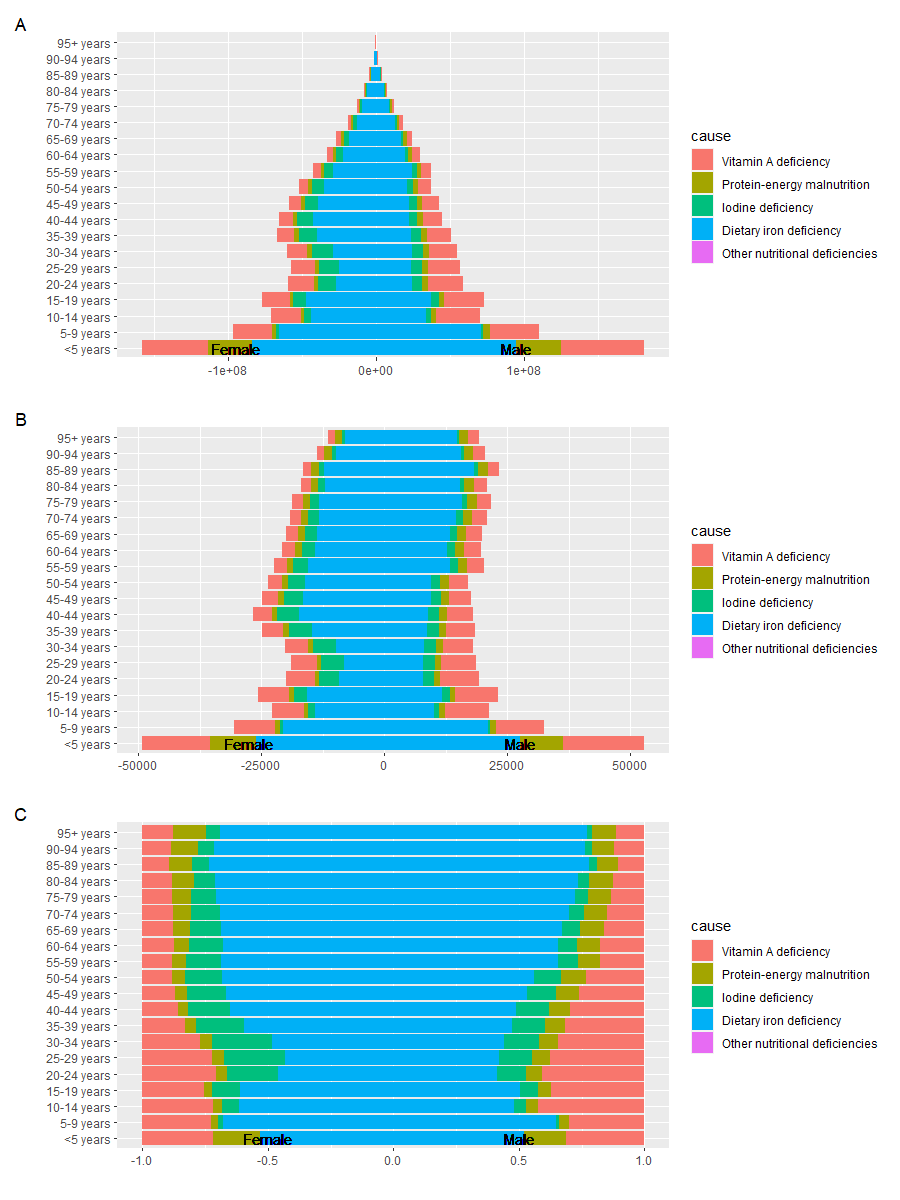
**

**Supplementary Figure 3.** Sex-differentiated Age-specific distribution of Causes for nutritional deficiencies. (A) Number of prevalence; (B) ASR- prevalence; (C) Proportion of prevalence. ASR: age-standardized rates.

**
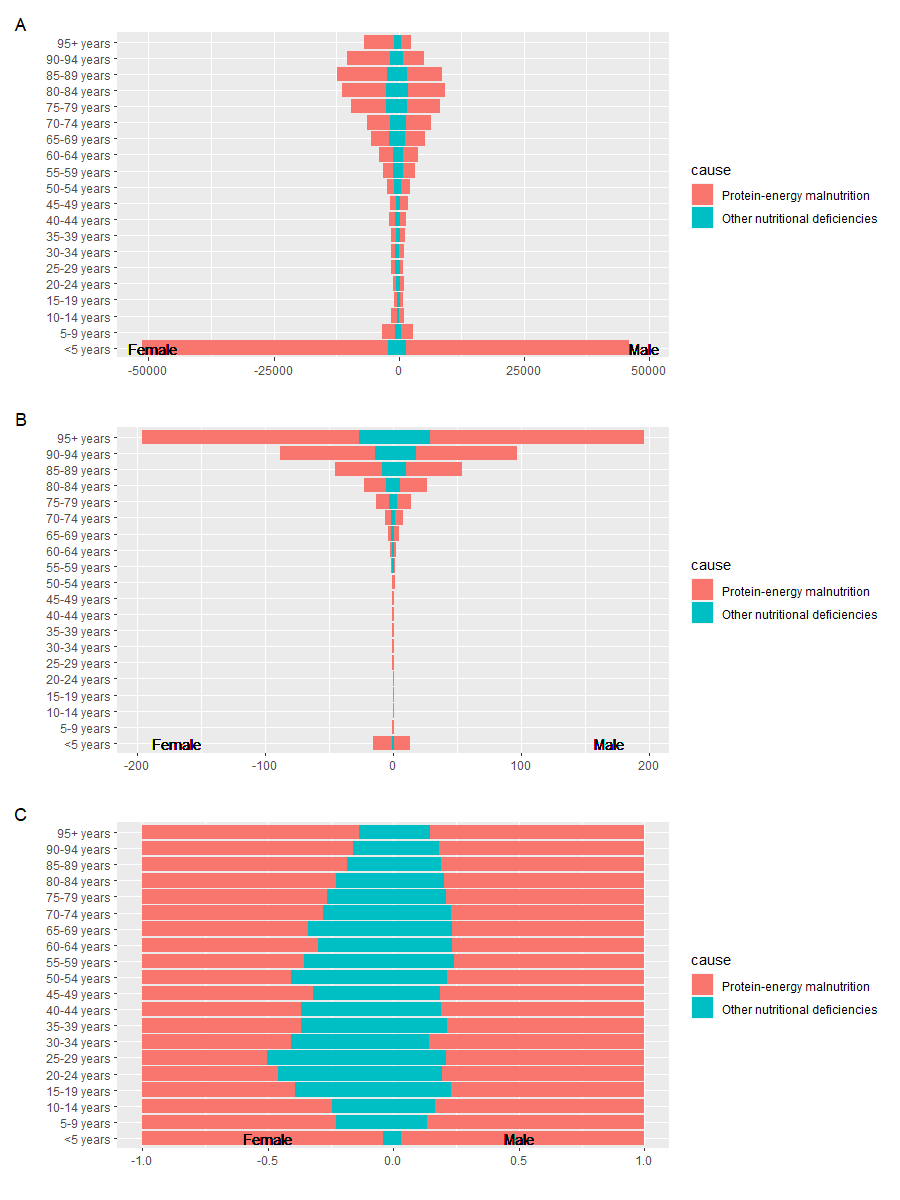
**

**Supplementary Figure 4.** Sex-differentiated Age-specific distribution of Causes for nutritional deficiencies. (A) Number of mortality; (B) ASR- mortality; (C) Proportion of mortality. ASR: age-standardized rates.


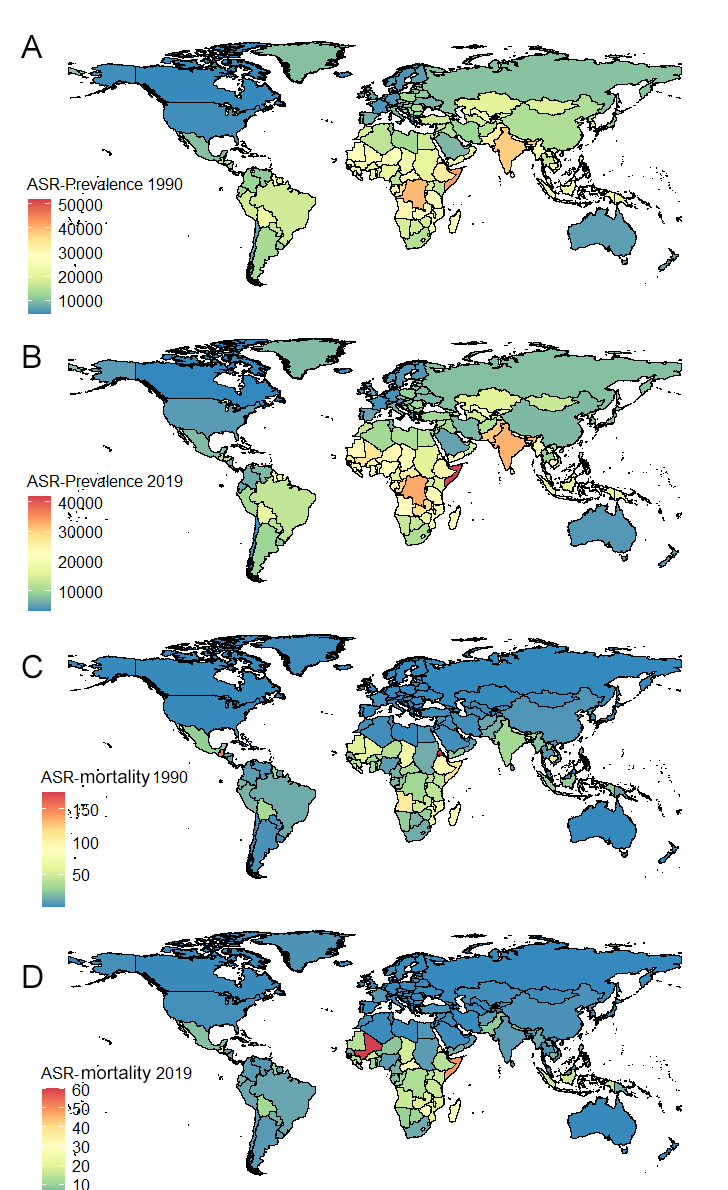


**Supplementary Figure 5** Global distribution maps for the burden of nutritional deficiencies in 204 countries and territories in 1990 and 2019. (A) ASR- prevalence in 1990; (B) ASR- prevalence in 2019; (C) ASR- mortality in 1990; (D) ASR- mortality in 2019. ASR: age-standardized rates.


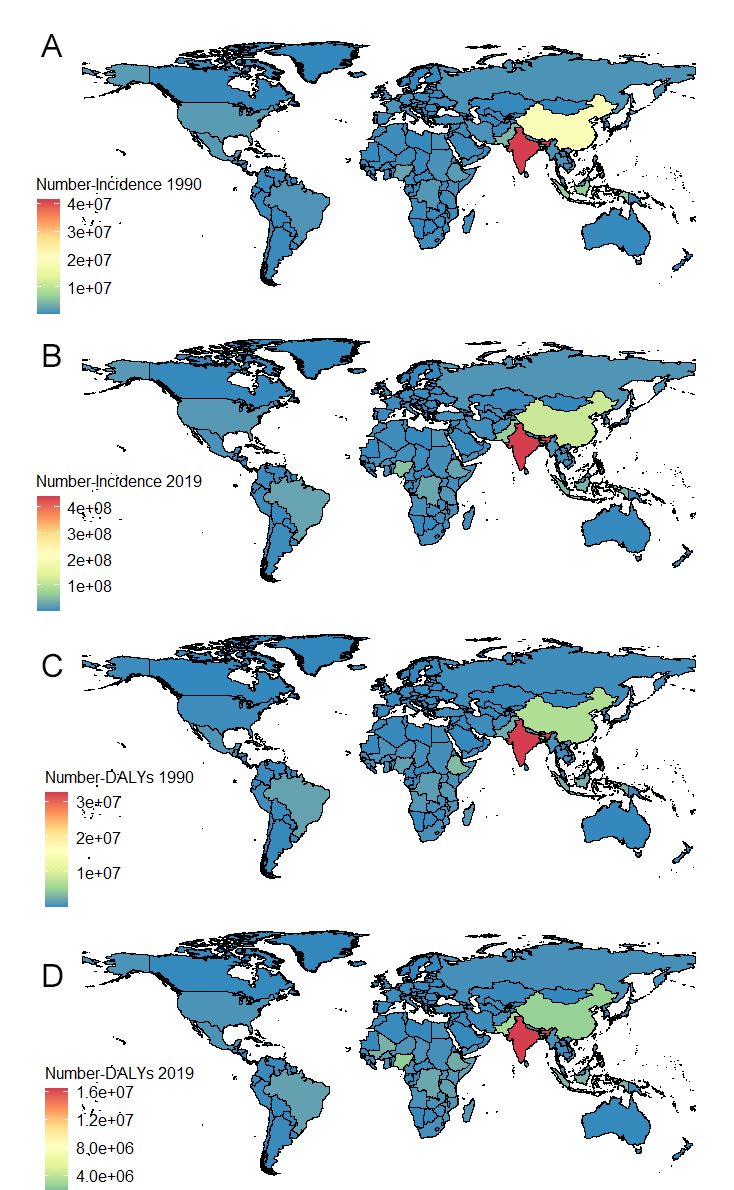


**Supplementary Figure 6** Global distribution maps for the burden of nutritional deficiencies in 204 countries and territories in 1990 and 2019. (A) Number- incidence in 1990; (B) Number- incidence in 2019; (C) Number- DALYs in 1990; (D) Number- DALYs in 2019. DALYs: disability-adjusted life years.


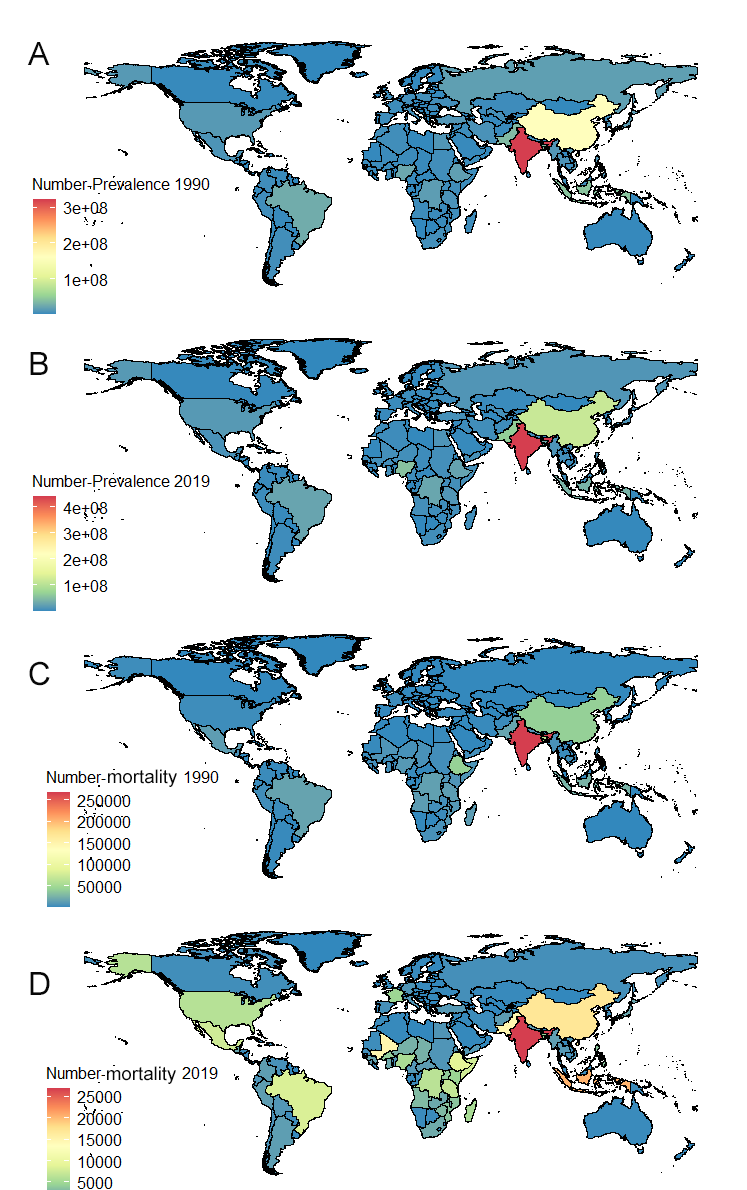


**Supplementary Figure 7** Global distribution maps for the burden of nutritional deficiencies in 204 countries and territories in 1990 and 2019. (A) Number- prevalence in 1990; (B) Number-prevalence in 2019; (C) Number- mortality in 1990; (D) Number- mortality in 2019.


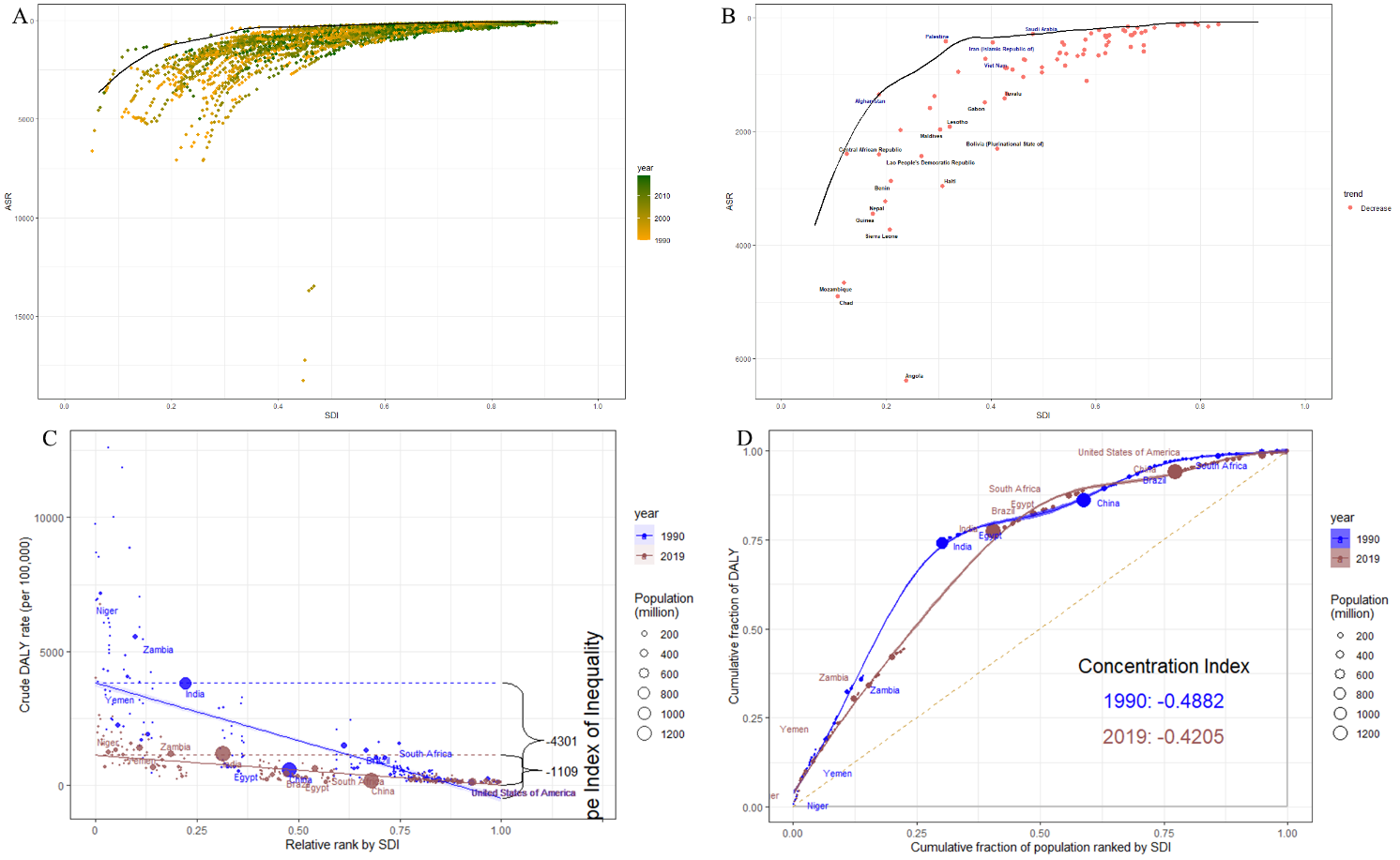


**Supplementary Figure 8** (A) Frontier analysis based on SDI and ASR-DALYs of nutritional deficiencies from 1990 to 2019. Color scale represents the years from 1990 depicted in orange to 2019 depicted in green. The frontier is delineated in solid black color. (B) Frontier analysis based on SDI and ASR-DALYs of nutritional deficiencies trend between 1990 and 2019. The frontier is delineated in solid black color; countries and territories are represented as dots. The top 15 countries with the largest effective difference (largest ASR-DALYs gap from the frontier) are labeled in black; the top 5 countries with the lowest effective difference with low SDI (<0.5) are labeled in blue. Red dots indicate a decrease in ASR-DALYs from 1990 to 2019. (C) Slope Indices Inequality for DALYs of nutritional deficiencies in 1990 and 2019 (the numbers adjacent to the brackets indicate the slopes.). (D) Concentration Indices for DALYs of nutritional deficiencies in 1990 and 2019 (Each country or region is represented by a solid dot, with larger dots indicating a higher population.). SDI: Sociodemographic Index; ASR: age-standardized rates; DALYs: disability-adjusted life years.


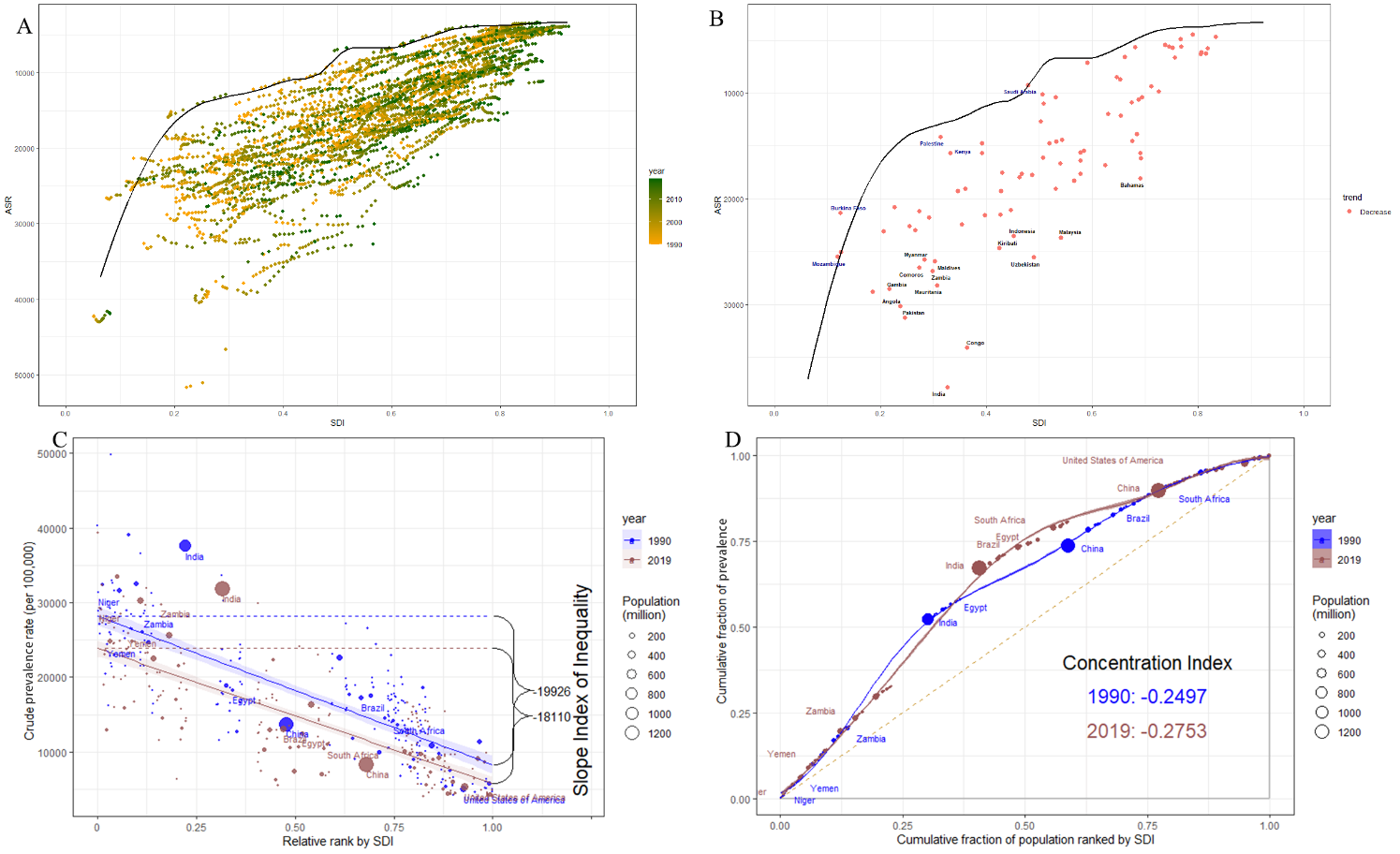


**Supplementary Figure 9** (A) Frontier analysis based on SDI and ASR-prevalence of nutritional deficiencies from 1990 to 2019. Color scale represents the years from 1990 depicted in orange to 2019 depicted in green. The frontier is delineated in solid black color. (B) Frontier analysis based on SDI and ASR- prevalence of nutritional deficiencies trend between 1990 and 2019. The frontier is delineated in solid black color; countries and territories are represented as dots. The top 15 countries with the largest effective difference (largest ASR- prevalence gap from the frontier) are labeled in black; the top 5 countries with the lowest effective difference with low SDI (<0.5) are labeled in blue. Red dots indicate a decrease in ASR- prevalence from 1990 to 2019. (C) Slope Indices Inequality for prevalence of nutritional deficiencies in 1990 and 2019 (the numbers adjacent to the brackets indicate the slopes.). (D) Concentration Indices for prevalence of nutritional deficiencies in 1990 and 2019 (Each country or region is represented by a solid dot, with larger dots indicating a higher population.). SDI: Sociodemographic Index; ASR: age-standardized rates.


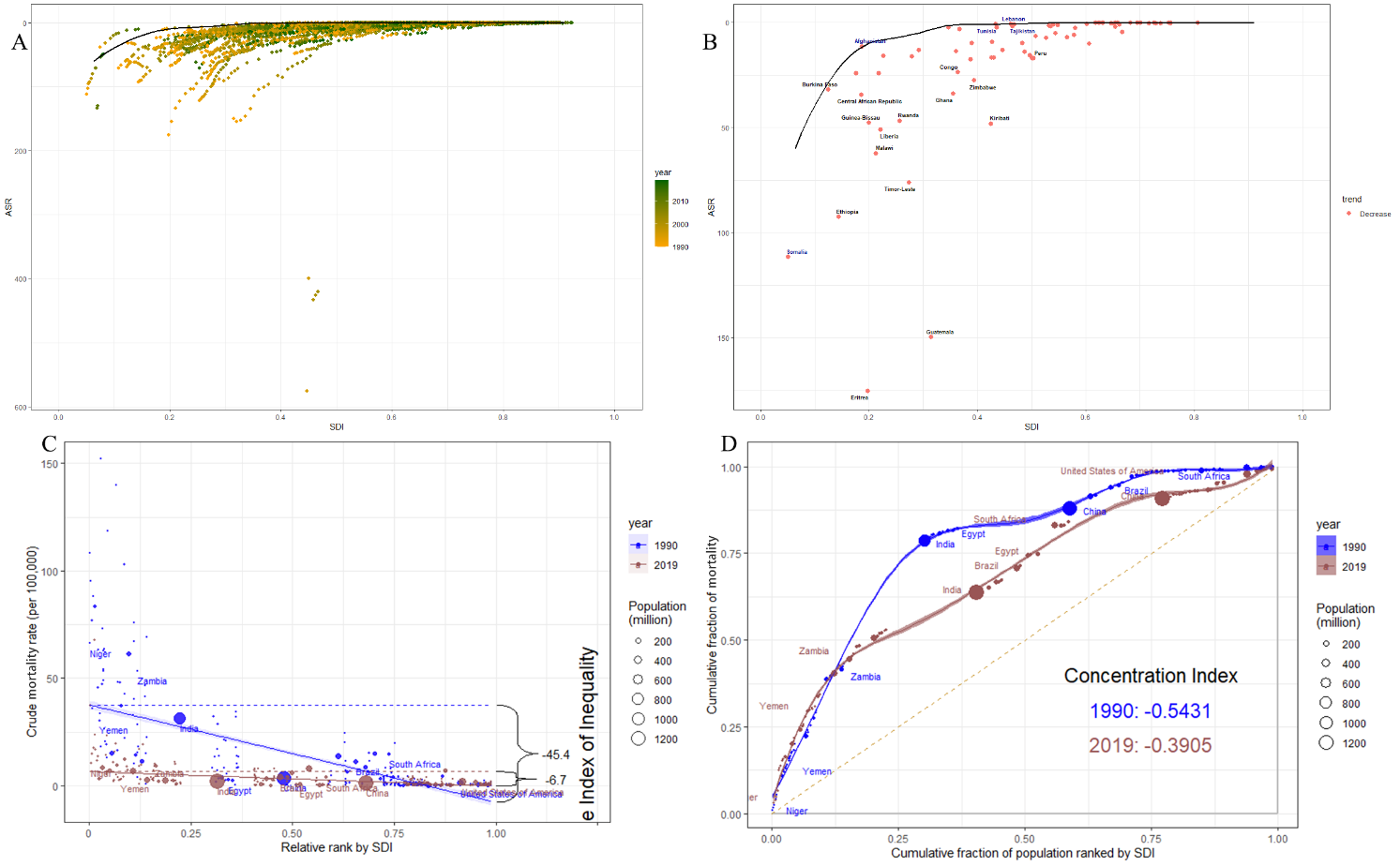


**Supplementary Figure 10** (A) Frontier analysis based on SDI and ASR- mortality of nutritional deficiencies from 1990 to 2019. Color scale represents the years from 1990 depicted in orange to 2019 depicted in green. The frontier is delineated in solid black color. (B) Frontier analysis based on SDI and ASR- mortality of nutritional deficiencies trend between 1990 and 2019. The frontier is delineated in solid black color; countries and territories are represented as dots. The top 15 countries with the largest effective difference (largest ASR- mortality gap from the frontier) are labeled in black; the top 5 countries with the lowest effective difference with low SDI (<0.5) are labeled in blue. Red dots indicate a decrease in ASR- prevalence from 1990 to 2019. (C) Slope Indices Inequality for mortality of nutritional deficiencies in 1990 and 2019 (the numbers adjacent to the brackets indicate the slopes.). (D) Concentration Indices for mortality of nutritional deficiencies in 1990 and 2019 (Each country or region is represented by a solid dot, with larger dots indicating a higher population.). SDI: Sociodemographic Index; ASR: age-standardized rates.

## Supplementary Tables

**Supplementary Table 1.** The number and ASR of prevalence and Mortality for nutritional deficiencies in 2019 and changing trends from 1990 to 2019.

|  | 2019 |  | 1990–2019 | 2019 | | | 1990–2019 | |
| --- | --- | --- | --- | --- | --- | --- | --- | --- |
|  | prevalence cases | ASPR per 100,000 (95% UI) | EAPC of ASPR (95% CI) | Mortality cases | ASMR per 100,000 (95% UI) | EAPC of ASMR (95% CI) | |  |
| Global | 1,281,205,444.40 (1,241,903,618.89 - 1,318,755,121.53) | 16,834.56 (16,336.72 - 17,312.89) | -0.13 (-11.95 to 13.29) | 251,577.35 (221,149.74 - 289,040.07) | 3.52 (3.09 - 4.06) | -4.92 (-16.14 to 7.8) | |  |
| Age (years) |  |  |  |  |  |  | |  |
| <5 years | 208,456,140.30 (205,130,694.00 - 211,832,193.10) | 31,448.81 (30,947.12 - 31,958.14) | -0.24 (-12.43 to 13.64) | 97,302.87 (75,647.54 - 125,680.60) | 14.68 (11.41 - 18.96) | -5.95 (-17.44 to 7.14) | |  |
| 5-9 years | 138,713,401.80 (133,424,351.90 - 143,978,257.10) | 21,187.20 (20,379.35 - 21,991.36) | 0.09 (-12.09 to 13.96) | 6,068.42 (4,818.04 - 7,470.29) | 0.93 (0.74 - 1.14) | -6.21 (-17.63 to 6.79) | |  |
| 10-14 years | 87,902,086.25 (82,694,292.32 - 93,060,755.48) | 13,687.93 (12,876.99 - 14,491.23) | 0.08 (-12.08 to 13.91) | 2,542.75 (2,067.20 - 3,049.46) | 0.40 (0.32 - 0.47) | -4.46 (-16.07 to 8.75) | |  |
| 15-19 years | 96,235,643.62 (91,534,456.18 - 101,161,055.50) | 15,533.38 (14,774.56 - 16,328.39) | 0.39 (-11.75 to 14.21) | 1,873.70 (1,581.72 - 2,190.51) | 0.30 (0.26 - 0.35) | -4.70 (-16.23 to 8.42) | |  |
| 20-24 years | 72,227,418.45 (67,410,070.92 - 77,800,955.34) | 12,035.01 (11,232.31 - 12,963.70) | -0.07 (-12.1 to 13.59) | 2,204.90 (1,864.82 - 2,598.00) | 0.37 (0.31 - 0.43) | -4.60 (-16.08 to 8.45) | |  |
| 25-29 years | 72,208,532.63 (66,596,451.18 - 78,245,720.52) | 11,926.03 (10,999.13 - 12,923.14) | -0.15 (-12.1 to 13.42) | 2,564.89 (2,145.11 - 3,100.43) | 0.42 (0.35 - 0.51) | -4.34 (-15.79 to 8.67) | |  |
| 30-34 years | 76,743,960.33 (71,281,681.30 - 82,839,585.32) | 12,753.83 (11,846.08 - 13,766.85) | -0.11 (-11.97 to 13.34) | 2,736.99 (2,332.05 - 3,222.47) | 0.45 (0.39 - 0.54) | -4.09 (-15.48 to 8.84) | |  |
| 35-39 years | 82,081,038.30 (77,435,611.43 - 86,989,702.88) | 15,172.77 (14,314.05 - 16,080.14) | 0.04 (-11.73 to 13.37) | 2,839.92 (2,441.61 - 3,293.02) | 0.52 (0.45 - 0.61) | -4.10 (-15.39 to 8.69) | |  |
| 40-44 years | 79,983,984.41 (75,898,976.88 - 84,365,090.22) | 16,209.36 (15,381.50 - 17,097.23) | 0.27 (-11.39 to 13.45) | 3,468.94 (3,002.43 - 4,092.51) | 0.70 (0.61 - 0.83) | -4.25 (-15.38 to 8.35) | |  |
| 45-49 years | 75,331,888.08 (71,546,362.88 - 79,639,906.76) | 15,899.48 (15,100.51 - 16,808.72) | 0.40 (-11.08 to 13.37) | 3,638.99 (3,195.17 - 4,219.70) | 0.77 (0.67 - 0.89) | -4.17 (-15.14 to 8.21) | |  |
| 50-54 years | 68,008,501.19 (64,405,812.21 - 71,825,254.32) | 15,569.11 (14,744.35 - 16,442.88) | 0.40 (-10.87 to 13.09) | 4,571.88 (4,006.37 - 5,320.86) | 1.05 (0.92 - 1.22) | -4.21 (-14.96 to 7.91) | |  |
| 55-59 years | 62,174,278.50 (59,079,412.82 - 65,568,620.28) | 16,758.00 (15,923.83 - 17,672.89) | 0.27 (-10.75 to 12.66) | 6,302.48 (5,574.74 - 7,284.88) | 1.70 (1.5 - 1.96) | -4.42 (-14.94 to 7.4) | |  |
| 60-64 years | 49,051,664.67 (46,421,403.09 - 51,679,689.25) | 15,694.76 (14,853.17 - 16,535.63) | 0.21 (-10.58 to 12.31) | 7,787.64 (6,927.47 - 8,934.22) | 2.49 (2.22 - 2.86) | -4.47 (-14.77 to 7.08) | |  |
| 65-69 years | 40,216,897.62 (38,099,538.77 - 42,550,503.53) | 15,552.76 (14,733.93 - 16,455.22) | 0.36 (-10.15 to 12.11) | 10,645.72 (9,514.22 - 12,151.40) | 4.12 (3.68 - 4.7) | -4.11 (-14.17 to 7.11) | |  |
| 70-74 years | 29,356,956.46 (27,713,294.06 - 30,987,707.73) | 15,691.60 (14,813.04 - 16,563.25) | 0.64 (-9.52 to 11.93) | 12,867.99 (11,764.36 - 14,198.41) | 6.88 (6.29 - 7.59) | -3.66 (-13.39 to 7.16) | |  |
| 75-79 years | 20,177,816.83 (18,995,788.49 - 21,403,665.74) | 15,881.33 (14,950.99 - 16,846.15) | 0.93 (-8.76 to 11.65) | 17,635.77 (16,115.78 - 19,385.24) | 13.88 (12.68 - 15.26) | -2.99 (-12.31 to 7.31) | |  |
| 80-84 years | 12,720,957.72 (11,788,762.17 - 13,559,445.31) | 15,068.24 (13,964.04 - 16,061.45) | 0.96 (-8.04 to 10.84) | 20,664.13 (18,526.77 - 22,597.63) | 24.48 (21.95 - 26.77) | -1.98 (-10.72 to 7.62) | |  |
| 85-89 years | 6,869,080.50 (6,363,985.89 - 7,357,729.03) | 15,797.93 (14,636.28 - 16,921.75) | 1.07 (-6.9 to 9.73) | 21,072.64 (18,083.35 - 23,214.31) | 48.46 (41.59 - 53.39) | -1.40 (-9.18 to 7.06) | |  |
| 90-94 years | 2,207,613.82 (2,011,958.27 - 2,417,379.56) | 13,095.67 (11,935.03 - 14,340.01) | 1.31 (-5.19 to 8.26) | 15,422.10 (12,393.51 - 17,196.70) | 91.48 (73.52 - 102.01) | -0.48 (-6.88 to 6.35) | |  |
| 95+ years | 537,582.93 (474,635.76 - 608,016.21) | 11,262.51 (9,943.75 - 12,738.10) | 1.65 (-2.99 to 6.51) | 9,364.63 (7,123.43 - 10,663.57) | 196.19 (149.24 - 223.4) | 0.99 (-3.63 to 5.83) | |  |
| Sex |  |  |  |  |  |  | |  |
| Male | 588,984,811.38 (570,341,189.29 - 606,915,628.92) | 15,499.28 (15,028.44 - 15,965.00) | -0.31 (-11.43 to 12.2) | 113,593.99 (99,257.28 - 129,011.81) | 3.45 (3.04 - 3.90) | -5.13 (-15.64 to 6.69) | |  |
| Female | 692,220,633.02 (670,677,682.67 - 714,738,838.81) | 18,221.90 (17,681.21 - 18,790.17) | 0.03 (-11.11 to 12.57) | 137,983.37 (118,650.81 - 165,844.07) | 3.64 (3.09 - 4.40) | -4.74 (-15.33 to 7.18) | |  |
| cause |  |  |  |  |  |  | |  |
| Vitamin A deficiency | 489,662,708.61 (469,006,373.61 - 512,234,291.26) | 6,955.65 (6,645.87 - 7,294.23) | -2.79(-14.28 to 10.23) | - | - | - | |  |
| Protein-energy malnutrition | 147,672,757.92 (130,405,923.71 - 167,471,359.50) | 2,006.38 (1,785.96 - 2,261.27) | 0.33(-11.55 to 13.81) | 212,242.12 (185,402.79 - 246,216.98) | 2.74 (2.40 - 3.18) | -5.00(-16.21 to 7.72) | |  |
| Iodine deficiency | 177,359,610.89 (144,480,601.25 - 219,049,834.88) | 2,215.54 (1,803.81 - 2,743.31) | -0.12(-11.96 to 13.31) | - | - | - | |  |
| Dietary iron deficiency | 1,069,047,090.63 (1,049,331,146.59 - 1,086,685,187.87) | 14,106.39 (13,850.72 - 14,342.09) | -0.17(-11.99 to 13.24) | - | - | - | |  |
| Other nutritional deficiencies | - | - | - | 39,335.24 (33,756.05 - 45,913.59) | 0.51 (0.44 - 0.59) | -4.42(-15.68 to 8.34) | |  |
| SDI |  |  |  |  |  |  | |  |
| High | 60,343,740.21 (56,977,084.53 - 63,578,450.09) | 5,703.92 (6,042.16 - 5,384.09) | -0.68 (-10.49 to 10.2) | 16,051.26 (13,335.89 - 17,672.37) | 0.68 (0.58 - 0.75) | 0.71 (-9.49 to 12.05) | |  |
| High-middle | 150,990,414.62 (144,581,169.65 - 157,510,301.65) | 10,489.65 (10,923.77 - 10,059.96) | -1.00 (-11.1 to 10.26) | 17,139.77 (15,214.94 - 18,564.01) | 0.98 (0.87 - 1.06) | -2.31 (-12.23 to 8.74) | |  |
| Middle | 326,162,191.19 (314,437,495.07 - 337,811,361.02) | 13,824.52 (14,289.24 - 13,350.27) | -0.66 (-11.26 to 11.21) | 54,704.80 (49,550.64 - 59,489.83) | 3.00 (2.68 - 3.29) | -3.29 (-13.42 to 8.03) | |  |
| Low-middle | 424,169,676.38 (411,314,355.95 - 437,640,007.16) | 24,557.50 (25,300.61 - 23,836.15) | -0.16 (-10.45 to 11.32) | 59,226.23 (50,936.47 - 70,361.76) | 4.51 (3.94 - 5.28) | -7.63 (-17.06 to 2.88) | |  |
| Low | 318,861,074.23 (309,609,816.94 - 328,346,346.89) | 27,852.28 (28,771.42 - 27,005.34) | 0.48 (-9.27 to 11.28) | 104,285.16 (83,825.01 - 130,286.64) | 11.56 (9.75 - 13.97) | -4.61 (-13.84 to 5.6) | |  |
| GBD region |  |  |  |  |  |  | |  |
| Central Asia | 16,651,376.71 (15,909,081.84 - 17,476,248.47) | 17,803.09 (18,685.02 - 17,009.46) | -0.31 (-7.6 to 7.54) | 263.48 (233.95 - 291.34) | 0.36 (0.32 - 0.40) | -4.77 (-11.67 to 2.66) | |  |
| East Asia | 123,473,541.29 (114,254,229.84 - 133,353,128.73) | 8,386.99 (9,058.06 - 7,760.76) | -1.94 (-12.02 to 9.3) | 17,431.25 (15,081.58 - 19,660.18) | 1.34 (1.15 - 1.52) | -7.27 (-16.68 to 3.2) | |  |
| South Asia | 553,740,246.25 (535,214,599.23 - 573,745,016.46) | 30,674.73 (31,782.90 - 29,648.49) | 0.03 (-10.28 to 11.53) | 47,207.62 (37,570.81 - 59,329.28) | 3.42 (2.75 - 4.28) | -7.86 (-17.27 to 2.63) | |  |
| Southeast Asia | 99,256,357.64 (95,060,801.38 - 103,552,175.67) | 14,731.23 (15,368.80 - 14,108.54) | -0.98 (-10.25 to 9.24) | 28,550.68 (25,275.50 - 31,485.45) | 6.30 (5.53 - 6.95) | -2.48 (-11.38 to 7.31) | |  |
| High-income Asia Pacific | 16,635,729.98 (15,179,881.09 - 18,234,603.27) | 8,882.28 (9,735.96 - 8,104.96) | -1.45 (-9.54 to 7.37) | 2,152.57 (1,757.47 - 2,384.85) | 0.39 (0.34 - 0.43) | 0.64 (-7.89 to 9.96) | |  |
| North Africa and Middle East | 69,097,781.07 (66,019,527.65 - 72,188,818.75) | 11,351.44 (11,859.24 - 10,845.74) | -0.69 (-9.8 to 9.34) | 5,877.22 (4,772.77 - 7,591.42) | 1.44 (1.20 - 1.80) | -4.58 (-13.25 to 4.97) | |  |
| Central Sub-Saharan Africa | 40,123,178.82 (37,132,931.97 - 43,394,476.13) | 30,501.59 (32,988.43 - 28,228.41) | 0.36 (-7.06 to 8.38) | 10,990.41 (7,837.27 - 15,444.85) | 13.86 (10.12 - 19.26) | -4.34 (-11.38 to 3.26) | |  |
| Eastern Sub-Saharan Africa | 94,368,668.49 (90,931,864.35 - 98,227,902.76) | 22,917.41 (23,854.62 - 22,082.78) | 0.38 (-8.3 to 9.89) | 51,564.24 (42,295.64 - 64,269.61) | 18.80 (16.09 - 22.03) | -4.76 (-12.91 to 4.15) | |  |
| Southern Sub-Saharan Africa | 10,067,307.94 (9,497,849.88 - 10,694,313.61) | 12,812.38 (13,610.36 - 12,087.65) | -0.22 (-7.32 to 7.42) | 5,644.67 (4,527.81 - 6,979.41) | 8.57 (7.08 - 10.41) | -1.52 (-8.44 to 5.93) | |  |
| Western Sub-Saharan Africa | 115,233,699.22 (110,396,495.48 - 119,969,743.20) | 25,253.23 (26,291.13 - 24,193.17) | 1.01 (-7.86 to 10.72) | 38,247.03 (28,849.77 - 50,401.00) | 9.15 (7.30 - 11.39) | -3.19 (-11.67 to 6.09) | |  |
| Andean Latin America | 6,775,969.09 (6,291,104.40 - 7,264,723.64) | 10,654.79 (11,423.32 - 9,892.37) | -1.33 (-8.07 to 5.9) | 2,929.17 (2,393.83 - 3,495.39) | 5.34 (4.38 - 6.38) | -4.52 (-10.92 to 2.35) | |  |
| Central Latin America | 19,346,252.43 (18,501,853.57 - 20,300,826.72) | 7,737.87 (8,119.67 - 7,400.14) | -0.73 (-8.99 to 8.28) | 12,092.68 (10,443.79 - 13,800.90) | 5.42 (4.68 - 6.19) | -4.02 (-11.86 to 4.52) | |  |
| Southern Latin America | 5,459,178.09 (4,870,872.73 - 6,130,827.13) | 8,178.16 (9,184.33 - 7,296.84) | -0.91 (-7.82 to 6.51) | 2,039.96 (1,804.39 - 2,233.84) | 2.43 (2.16 - 2.66) | -0.55 (-7.52 to 6.95) | |  |
| Tropical Latin America | 29,126,581.90 (26,388,448.53 - 31,954,189.30) | 13,026.48 (14,291.08 - 11,801.88) | -0.56 (-8.73 to 8.34) | 8,524.68 (7,544.00 - 9,191.86) | 3.97 (3.51 - 4.30) | -3.61 (-11.43 to 4.91) | |  |
| High-income North America | 18,746,065.93 (16,814,814.45 - 20,882,181.27) | 5,142.10 (5,728.04 - 4,612.35) | 0.55 (-8.31 to 10.26) | 6,444.95 (5,534.21 - 6,947.01) | 0.89 (0.78 - 0.95) | 1.05 (-8.09 to 11.1) | |  |
| Caribbean | 7,582,382.22 (7,216,575.38 - 7,946,539.60) | 16,075.62 (16,847.68 - 15,300.06) | -0.09 (-6.71 to 7) | 1,862.85 (1,432.77 - 2,431.41) | 4.13 (3.14 - 5.53) | -3.46 (-9.85 to 3.38) | |  |
| Australasia | 1,333,389.59 (1,128,416.17 - 1,552,324.89) | 4,587.81 (5,341.10 - 3,882.55) | -0.69 (-6.68 to 5.67) | 179.05 (148.53 - 204.26) | 0.31 (0.26 - 0.35) | 0.41 (-5.88 to 7.12) | |  |
| Oceania | 2,620,205.99 (2,444,891.93 - 2,802,661.04) | 19,735.76 (21,110.03 - 18,415.27) | 0.69 (-4.38 to 6.02) | 308.16 (228.92 - 427.65) | 4.84 (3.74 - 6.41) | -1.37 (-6.03 to 3.51) | |  |
| Central Europe | 9,911,840.30 (9,290,903.57 - 10,598,800.94) | 8,677.57 (9,278.99 - 8,133.96) | -1.22 (-8.84 to 7.03) | 426.37 (370.57 - 480.97) | 0.22 (0.19 - 0.25) | 2.12 (-5.88 to 10.81) | |  |
| Eastern Europe | 18,855,099.56 (17,090,121.84 - 20,799,958.95) | 8,979.87 (9,906.12 - 8,139.29) | -1.08 (-9.39 to 8) | 653.06 (584.27 - 737.08) | 0.23 (0.20 - 0.25) | -2.60 (-10.84 to 6.41) | |  |
| Western Europe | 22,800,591.88 (21,126,959.62 - 24,680,897.66) | 5,225.81 (5,656.77 - 4,842.22) | -0.90 (-9.86 to 8.95) | 8,187.27 (6,587.93 - 9,418.66) | 0.67 (0.55 - 0.77) | 1.40 (-8.13 to 11.91) | |  |
